# Inhibition of SENP6 restrains cerebral ischemia-reperfusion injury by regulating Annexin-A1 nuclear translocation-associated neuronal apoptosis

**DOI:** 10.7150/thno.60277

**Published:** 2021-06-01

**Authors:** Qian Xia, Meng Mao, Zhen Zeng, Zhenzhao Luo, Yin Zhao, Jing Shi, Xing Li

**Affiliations:** 1 Department of Anesthesiology, Tongji Hospital, Tongji Medical College, Huazhong University of Science and Technology, Wuhan 430030, Hubei Province, China.; 2 Department of Neurobiology, School of Basic Medicine, Tongji Medical College, Huazhong University of Science and Technology, Wuhan 430030, Hubei Province, China.; 3 Department of Medical Laboratory, The Central Hospital of Wuhan, Tongji Medical College, Huazhong University of Science and Technology, Wuhan 430030, Hubei Province, China.; 4 Department of Ophthalmology, Tongji Hospital, Tongji Medical College, Huazhong University of Science and Technology, Wuhan 430030, Hubei Province, China.

**Keywords:** SENP6, Annexin-A1, deSUMOylation, cerebral ischemia-reperfusion injury, nuclear translocation, neuronal apoptosis

## Abstract

**Rationale:** Annexin-A1 (ANXA1) has previously been proposed to play a crucial role in neuronal apoptosis during ischemic stroke injury. Our recent study demonstrated that ANXA1 was modified by SUMOylation, and that this modification was greatly weakened after cerebral ischemia, but its effect on neuronal death and the underlying mechanism have not been fully elucidated.

**Methods:** Mice subjected to middle cerebral artery occlusion were established as the animal model and primary cultured neurons treated with oxygen-glucose deprivation and reperfusion was established as the cell model of ischemic stroke. The Ni^2+^-NTA agarose affinity pull-down assay was carried out to determine the SUMOylation level of ANXA1. Co-immunoprecipitation assays was utilized to explore the protein interaction. Immunoblot analysis, quantitative real-time PCR, Luciferase reporter assay were performed to identify the regulatory mechanism. LDH release and TUNEL staining was performed to investigate the neuronal cytotoxicity and apoptosis, respectively.

**Results:** In this study, we identified the deSUMOylating enzyme sentrin/SUMO-specific protease 6 (SENP6) as a negative regulator of ANXA1 SUMOylation. Notably, we found that SENP6-mediated deSUMOylation of ANXA1 induced its nuclear translocation and triggered neuronal apoptosis during cerebral ischemic injury. A mechanistic study demonstrated that SENP6-mediated deSUMOylation of ANXA1 promoted TRPM7- and PKC-dependent phosphorylation of ANXA1. Furthermore, blocking the deSUMOylation of ANXA1 mediated by SENP6 inhibited the transcriptional activity of p53, decreased Bid expression, suppressed caspase-3 pathway activation and reduced the apoptosis of primary neurons subjected to oxygen-glucose deprivation and reperfusion. More importantly, SENP6 inhibition by overexpression of a SENP6 catalytic mutant in neurons resulted in significant improvement in neurological function in the mouse model of ischemic stroke.

**Conclusions:** Taken together, the results of this study identified a previously unidentified function of SENP6 in neuronal apoptosis and strongly indicated that SENP6 inhibition may provide therapeutic benefits for cerebral ischemia.

## Introduction

Cerebral ischemia has long been recognized as a common and serious disease with high morbidity, disability and mortality 1-3. Management focuses on rapid reperfusion with intravenously administered tissue-plasminogen activator (tPA), also known as thrombolytic therapy, and mechanical thrombectomy, which both are well accepted to restore brain function for acute cerebral ischemia during a limited treatment window after stroke 4, 5. Other important strategies include preventing recurrent stroke, maximizing the rehabilitation achieved and preventing or treating complications. To date, there is no safer or more effective treatment strategy when patients miss the best treatment time. Apoptosis is one of the fundamental mechanisms of cell death that occurs during ischemic brain injury, and neurons are particularly vulnerable because of their high metabolic demand 6. Thus, targeted inhibition of proapoptotic factors would provide a beneficial therapeutic strategy for ischemic stroke.

Annexin A1 (ANXA1) is a well-recognized 37 kDa member of the annexin superfamily involved in the regulation of multiple cellular functions of diverse cell types 7-11. There is also overwhelming evidence for a role of ANXA1 in neuronal apoptosis during cerebral ischemia 12, 13. A previous study reported that importin β-dependent nuclear ANXA1 translocation is involved in oxygen-glucose deprivation and reperfusion (OGD/R)-induced neuronal apoptosis. As a cofactor, ANXA1 binds p53 in the nucleus and upregulates p53 transcriptional activity, thereby subsequently promoting proapoptotic Bid expression and caspase-3 apoptosis pathway activation, which ultimately leads to neuronal apoptosis after ischemic stroke 14. The results of an extensive study indicated that the biological functions of ANXA1 are tightly regulated by posttranslational modifications (PTMs), such as phosphorylation 15. Our previously study revealed that ANXA1 was modified by the small ubiquitin-related modifier (SUMO) protein, a process termed SUMOylation, and this modification was greatly weakened after cerebral ischemia 16. However, the key enzymes that regulate the SUMOylation status of ANXA1 and its effect on neuronal death after cerebral ischemia remain poorly understood.

SUMOylation is a widespread and tightly controlled posttranslational modification that regulates various protein functions, such as subcellular localization, stability, and protein-protein interactions 17-20. Previous data suggested that the conjugation of SUMO2 and SUMO3 is predominantly induced as an intracellular protective response to cellular stresses, such as hypoxia and inflammatory stimuli 21. There are also a series of reports showing a neuroprotective role of SUMOylation in brain lesions caused by ischemia-reperfusion injury 22. SUMOylation is a reversible and dynamic process in that it can be readily reversed by a family of specific isopeptidases referred to as sentrin/SUMO-specific proteases (SENPs), and six SENPs (SENP1-3, 5-7) have been identified in mammalian cells 23, 24. SENP3-mediated deSUMOylation of Drp1 promotes cell death following ischemia, but SENP1 could protect neurons from apoptotic death during transient brain ischemia and reperfusion 25, 26. Nevertheless, which enzyme of the SENP family regulates the SUMOylation level of ANXA1 has been rarely investigated.

In the present study, we provide experimental evidence that SENP6 mediates the deSUMOylation of ANXA1 and that the interaction between SENP6 and ANXA1 is increased in neurons subjected to OGD/R. The downregulation of ANXA1 SUMOylation resulted in an increase in its phosphorylation level, which in turn promoted its nuclear translocation, followed by activation of proapoptotic Bid gene expression and the caspase-3 apoptosis pathway, ultimately leading to neuronal apoptotic death after cerebral ischemia-reperfusion injury. Importantly, inhibition of SENP6-mediated deSUMOylation of ANXA1 significantly improved neurological function after cerebral ischemia. The results of this study show that SENP6 inhibition may provide a novel and potential therapeutic strategy for treating cerebral ischemia.

## Materials and Methods

### Animals

C57BL/6J male mice (weighing 22-25 g, aged 8 weeks) were obtained from Beijing Vital River Laboratory Animal Corp. Ltd. Mice were housed in special breeding box (five per cage) which was placed in a quiet room. The breeding environment was kept at a 12-12 h light-dark cycle, controlled temperature (about 22 °C), 40%-60% relative humidity with water and food ad libitum. After surgical operations and management, the housing environment still maintains the above standards. All animal experiments were approved by the Ethics Committee for Animal Experimentation of Huazhong University of Science and Technology (Wuhan, China). The study was conducted according the IMPROVE guidelines 27. The protocols and details of this report were in accordance with the Animal research: reporting of *in vivo* experiments (ARRIVE) guidelines 28. The sample size was obtained by analyzing pre-experimental data with PASS (power analysis & sample size) software using a significance level of α = 0.05 with 80% power to detect statistical differences. For animal studies, the number of animals for each group was predetermined according to numbers reported in published studies or our prior experiment and the accurate animal numbers are given in figure legends. Randomization was used in all experiments. Investigators were blind to treatment group when assessing the outcome.

### Reagents and Antibodies

The Ni^2+^-NTA agarose were obtained from QIAGEN (Dusseldorf, Germany) and Protein A+G Agarose beads were purchased from Beyotime Biotechnology (Shanghai, China). N-ethylmaleimide (NEM) were purchased from Sigma-Aldrich (St. Louis, MO). The complete protease inhibitor cocktail, the PhosSTOP phosphatase inhibitor cocktail and the *In Situ* Cell Death Detection Kit was purchased from Roche (Basel, Switzerland). Primary antibodies used in this research were listed below: anti-annexin A1 (sc-12740, 1:1000), anti-HA (sc-7392, 1:1000), anti-Flag (sc-166355, 1:1000), anti-α-tubulin (sc-100585, 1:2000), anti-β-actin (sc-47778, 1:2000), anti-PKC (sc-17769, 1:1000), anti-Bid (sc-11423, 1:1000) were purchased from Santa Cruz Biotechnology (Dallas, TX); anti-SUMO2/3 (#4971, 1:1000), anti-Myc (#2276, 1:1000), anti-Histone H3 (#4499, 1:2000), anti-cleaved caspase-3 (9664, 1:1000), anti-cleaved PARP (5625, 1:1000), anti-cleaved caspase-9 (#20750, 1:1000) were obtained from Cell Signaling Technology (Danvers, MA); anti-SENP6 (HPA024376, 1:1000), anti-His (SAB1306085, 1:1000) were purchased from Sigma-Aldrich, and anti-TRPM7 (ab232455, 1:500) were purchased from Abcam (Cambridge, UK). All other general reagents were purchased from commercial suppliers and used as received.

### Transient focal cerebral ischemia

Focal cerebral ischemia was induced by transient occlusion of the middle cerebral artery using intraluminal filament technique. Briefly, the mice were anaesthetized with chloral hydrate (350 mg/kg, i.p.). The whole operation was conducted on a thermostatic blanket (Harvard instrument, Holliston, MA) which were used to maintain the rectal temperature at about 37±0.5°C. The left common carotid artery (CCA), external carotid artery (ECA) and internal carotid artery (ICA) were exposed. Ligation of the CCA was performed with surgical nylon monofilament near the distal end of the CCA and ligation of the ECA was conducted at two positions at the end of ECA and near ICA and ECA bifurcations. Then, between the two ECA ligatures, we made a small incision. Nylon filament about two diameter lengths was softly intercalated into the ICA through the ECA stump and pushed into the anterior cerebral artery until slight resistance was felt which was approximately the location of the mark place. Successful occlusion was verified by a laser Doppler flowmetry (PeriFlux System 5000, PERIMED, Stockholm, Sweden). After 60 minutes of ischemia, draw out the filament and following by applying appropriate amoxicillin to the surgical areas to prevent infection. The sham mice were conducted with the surgical operating but no operation to insert an embolus.

### Cell culture, transfection and OGD/R procedure

Primary neurons were cultured as previously described 12. In brief, primary neurons were isolated from C57BL/6 mouse embryos (E18) under a dissection microscope, washed with D-Hank's solution under sterile conditions and seeded at a density of 1 × 10^6^ cells/cm^2^ on plates coated with poly-L-lysine (50 mg/mL) (Sigma-Aldrich). The cells were grown in Neurobasal medium supplemented with 2% B27 (Gibco, Gaithersburg, MD), 1% glutamine, and 1% penicillin/streptomycin (ThermoFisher Scientific, Waltham, MA) at 37°C in a humidified incubator in air containing 5% CO_2_. The purity of the neurons was confirmed by NeuN immunofluorescence at 5 days after plating; more than 95% of the cells in the culture were positive for NeuN.

HEK293T and Neuro-2a (N2a) cells were purchased from American Type Culture Collection (ATCC). Cells were grown in DMEM supplemented with 10% FBS and 1% penicillin-streptomycin (Sigma-Aldrich) at 37 °C in a humidified 5% CO_2_-containing atmosphere. Confluent cell layers were split three times per week. Transfections of the plasmids into HEK293T cells were performed by using Lipofectamine 3000 (Invitrogen, Carlsbad, CA) when the cells were 80-90% confluent, following the manufacturer's instructions. OGD/R was performed as we previously described 29. Briefly, the cell culture medium was replaced with glucose-free DMEM (Gibco) pre-heated to 37°C, after which the cells were transferred to an anaerobic incubator with a 5% CO_2_ and 95% N_2_ atmosphere at 37°C for 1 h. After washing the cultures with DMEM three times, the cultures were re-oxygenated under normoxic conditions for 24 h before they were collected for assays.

### Protein extraction and preparation

Protein extraction and subcellular separation were conducted as described previously 14. To prepare whole-cell lysates, the radioimmunoprecipitation assay (RIPA) lysate (Beyotime Biotechnology) were used to split the cells, while the RIPA was supplemented with 5 mg/ml cOmplete™ protease inhibitor cocktail (Roche). The NE-PER™ nuclear and cytoplasmic extraction kit (ThermoFisher Scientific) was used to separate the cytoplasmic and nuclear isolates based on manufacturer's instructions. The protein concentration of the extracts was determined by BCA protein assay kit (Beyotime Biotechnology).

### Ni^2+^-NTA affinity purification

As described previously, nickel nitrilotriacetic acid (Ni^2+^-NTA) pull-down was used to purify the SUMOylation of ANXA1 in the case of denaturation 16. In brief, HEK293T cells were first washed with cold PBS twice, following by treating with 800 ml Ni^2+^-NTA denatured buffer (10 mM Tris; 20 mM NEM; 6 M Gu-HCl and 100 mM NaH_2_PO_4_ pH 8.0). The sample was cleared via centrifugation (15 000×g, 10 min, 4 °C) after the DNA was cut by ultrasound treatment (2 × 20 s). The supernatant was composited with 50 ml of pre-washed Ni^2+^-NTA agarose (Qiagen) and hatched on a shaker at 4°C for 3 hours. Then 1 ml Ni^2+^-NTA washing buffer (0.1% Triton X-100; 100 mM NaH_2_PO_4_; 10 mM Tris/HCl, pH 6.3; 8 M Urea) was used to wash the beads which were finally eluted in 50 ml 2 × SDS-PAGE loading buffer supplemented with 200 mM imidazole at 95°C for 5 min. Finally, binding proteins were conducted via immunoblot analysis.

### LDH release and cell survival assays

LDH release was analysed using a LDH Cytotoxicity Assay Kit (Beyotime Biotechnology) according to the manufacturer's instructions. Three independent experiments were performed. For the survival assays, cells that had undergone the indicated treatments were fixed in 4% formaldehyde for 15 min and permeabilized with 0.1% Triton X-100. Thereafter, the neurons were incubated with 50 ml/well TUNEL reaction mixture for 1 h and with 1 mg/ml of DAPI (Sigma-Aldrich) for 10 min at 37°C. The cells were then examined and counted immediately under fluorescence microscopy (IX73, Olympus, Tokyo, Japan).

### Immunoprecipitation and immunoblot analysis

Immunoprecipitation was performed as described previously 30. The immunoprecipitation (IP) buffer supplemented with a protease inhibitor mixture (Beyotime Biotechnology) or NEM (*N-ethylmaleimide*, Sigma-Aldrich) was used to lyse cells. Simply, anti-Myc, anti-HA, anti-SENP6 and anti-ANXA1 antibodies were added into some of the cell lysates and incubated for overnight on a shaker at 4 °C, and then incubated with protein A/G plus agarose (Santa Cruz) at 4°C for 4 hours. Wash buffer were used to the samples for three times. 2× SDS-PAGE loading buffer were added to lysates and followed by boiling at 95 °C for 5min, and the protein expression was analyzed by immunoblot analysis.

For immunoblot detection, sodium dodecyl sulfate-polyacrylamide gel electrophoresis (SDS-PAGE) was used to separate the samples which were transferred to polyvinylidene fluoride (PVDF) membrane (Millipore, Schwalbach, Germany). Next, the PVDF membrane was blocked with 5% skim milk for 60 min at room temperature and incubated overnight with primary antibody at 4°C. The second day, the PVDF membrane were washed with TBST for three times, and then incubated with secondary antibodies for 60 min. The target protein was visualized by incubation with an enhanced chemiluminescence (ECL, Pierce, Rockford, IL) kit referring to the manufacturer's instructions. ImageJ software (NIH, Baltimore, MD) were used to quantify the target protein levels after standardizing internal parameter.

### Immunofluorescence staining

The cultured N2a cells fixed in 4 % paraformaldehyde were thoroughly rinsed with PBS three times and treated with 10 % Triton X-100 for 10 min to rupture the cell membranes. The fixed cells were then blocked with 10 % donkey serum. One hour later, the coverslips were incubated overnight at 4 °C with the following primary antibodies: anti-HA (sc-7392, 1:200, Santa Cruz Biotechnology) or anti-annexin A1 (sc-12740, 1:200, Santa Cruz Biotechnology). After washing three times, the fixed coverslips were incubated with Alexa Fluor 488-conjugated AffiniPure goat anti-mouse IgG (H+L) (Jackson Immuno Research, West Grove, PA) for 60 min at room temperature. Finally, the fluorescence was captured by fluorescence microscopy (IX73, Olympus). Regional fluorescence intensities were quantified using Image Pro Plus software. To measure regional intensities, small circles within the cytoplasmic or nuclear regions of each cell were selected using the elliptical marquee tool. The intensity within each circle was obtained using the histogram function for each color channel, which was selected using the layers/channels palette. The values were recorded, for evaluation of the ratio of the nuclear to cytoplasmic intensity; we examined up to 10 independent fields of cells and scored at least 100 cells for each measurement.

### RNA extraction, reverse transcription, and quantitative real-time PCR (qRT-PCR)

According to reagent supplies user manual, total RNA was extracted from cultured cells by TRIzol reagent (Invitrogen) and the concentration was measured by Spectrophotometry. Next, according to the instruction of ReverTra Ace-α-TM First Strand cDNA Synthesis Kit (Toyobo, Osaka, Japan), 1 mg of total RNA was reverse transcription into cDNA. As recommended by the manufacturer, qRT-PCR was conducted by StepOnePlus^TM^ Real-Time PCR System with SYBR Green PCR Master Mix (Applied Biosystems, Foster City, CA). We normalized the relative gene expression to the *β-actin* mRNA levels, following by 2^-ΔΔCt^ method to analyze gene expression. The primers used were as follows: *Senp6*, 5′-ATG CAG ACA AAG ATG GGG CA-3′ (forward) and 5′-CAG TCT TGC TCC GCC TTA CA-3′ (reverse); *Bid*, 5′-GCC TGT CGG AGG AAG ACA AA-3′ (forward) and 5′-GTG GAA GAC ATC ACG GAG CA-3′ (reverse); *β-actin*, 5′-GGC TGT ATT CCC CTC CAT CG-3′ (forward) and 5′-CCA GTT GGT AAC AAT GCC ATG T-3′ (reverse).

### Luciferase reporter assay

HEK293T cells were transfected with PG13-Luc (with p53 binding cis-element) and HA-ANXA1-WT, HA-ANXA1-3KR, HA-ANXA1-SUMO2, or Myc-SENP6-WT, Myc-SENP6-C1030S, SENP6 shRNA with HA-ANXA1 present or absent based on the experimental strategy. Each transfection included the pRL-Renilla as control. After incubation for 48 h at 37 °C, the cell lysate was collected for luciferase assay using a Dual-Luciferase kit (Promega, Madison, WI). The firefly-luminescence results were normalized to the Renilla luminescence for internal control of transfection efficiency. The experiments were performed in triplicate.

### Plasmids construction

The full-length DNA segment ANXA1 coding sequence was amplified by PCR, and cloned into HA-tagged pcDNA 3.0 (HA-ANXA1). The His-tagged SUMO2 was generated via amplifying the coding sequence and cloned into His-pcDNA 3.0 (His-SUMO2). The Flag-tagged Ubc9 was generated using PCR, followed by cloning the full-length cDNA into p3xFLAG CMV14 (Flag-Ubc9). The Myc-tagged SENP1, SENP2, SENP3, SENP5, SENP6, SENP7 were generated via cloning the indicated cDNA into pcDNA 3.0. For site-directed mutagenesis, the subsequent mutants ANXA1-3KR (ANXA1-K113/161/257R, Lysine-to-Arginine) and SENP6-C1030S (Cysteine-to-Serine) were constructed using homologous recombination via Trelief SoSoo Cloning Kit Ver.2 reference to product specification (TSINGKE, Beijing, China). Human SENP6 shRNA plasmids for HEK293T cell transfected were purchased from GenePharma (Suzhou, China). The target sequence for human SENP6 shRNA #1 was 5′-GAC AGA ACT AAC AGA AGA GAA-3′, and the target sequence for human SENP6 shRNA #2 was 5′-CAC AGG ATT AAC AAC CAA GAA-3′. All constructs were confirmed by DNA sequencing analysis (performed by Sangon Biotechnology, Shanghai, China).

### Viral vectors production and transduction

Adenoviruses encompassing the Myc-tagged wild type ANXA1 (ANXA1-WT), ANXA1 triple-mutant (ANXA1-3KR) and constitutive SUMOylated mimic of ANXA1 (ANXA1-SUMO2) were generated by Vigene Biosciences (Jinan, China). The shRNA duplexes targeting mouse SENP6 and ANXA1 were constructed as we previously reported 16. The sequences of shRNAs are designed and verified as follows: mSENP6-#1, 5′-GGG CAA ATC TAC TCA GTG TAG-3′; mSENP6-#2, 5′-AAG AAA GTG AAG GAG ATG CAG-3′; mANXA1, 5′-GCC TCA CAA CCA TCG TGA AGT-3′. Primary cultured neurons or N2a cells were infected with serially diluted concentrations of recombinant adenovirus. The optimal multiplicity of infection was determined to be 50:1 to 100:1. After the adenovirus infection for 48 h, cells were subjected to OGD/R and (or) other treatments.

The lentivirus for overexpressing WT SENP6 (LV-CamKⅡa-SENP6-WT), SENP6 (LV-CamKⅡa-SENP6-C1030S) and the control lentivirus (LV-CamKⅡa-Vector) were designed and obtained commercially from Genechem (Shanghai, China). Two weeks before MCAO surgery, mice received a stereotactic injection of the lentivirus. The injection was performed as previously described 16. Briefly, mice were anesthetized with 5% chloral hydrate (350 mg/kg, i.p.), and placed in a stereotaxic apparatus (Stoelting, Wood Dale, IL). A burr hole was used to perforate the skull, and a virus solution of 0.5-1.0 ml per injection site was injected into the hippocampus CA1 region, cerebral cortex and striatum of the left hemisphere with a stepper-motorized microsyringe (Hamilton, Reno, NV). The needle was kept in place for an additional 5 min to prevent reflux and was then removed slowly. Injections into the hippocampus CA1 were at coordinates anteroposterior (AP) -2.00 mm, lateral (L) -1.55 mm and dorsoventral (DV) -1.55 mm, cerebral cortex were at coordinates anteroposterior (AP) 0.00 mm, lateral (L) -2.05 mm and dorsoventral (DV) -1.50 mm, and striatum were at coordinates anteroposterior (AP) 0.00 mm, lateral (L) -2.05 mm and dorsoventral (DV) -3.50 mm relative to the bregma and dura surface.

### TTC Staining

At 24 h after MCAO surgery, the mice were sacrificed and the brain were quickly extracted, following by freezing for five minutes at -20 °C. The mouse brain matrix (RWD Life Science, Shenzhen, China) was used to cut the entire brain into 6 consecutive 2-millimeter-thick coronal slices from the frontal tip, following by dyeing with 1% TTC (Sigma-Aldrich). The whole incubation process lasted 20 minutes at 37°C. Then the brain slices were steeped with 4% paraformaldehyde for immobilization. The white region was defined as the ischemic zone, while the dark red regions corresponding to normal tissue. The infarct areas in each section were calculated by ImageJ analysis software (NIH, Baltimore, MD). A correction for edema was made according to the following formula: infarct area × (area contralateral hemisphere/area ipsilateral hemisphere) 31. Cerebral infarct volume was measured as a percentage of the total contralateral hemisphere, as calculated with the following formula: total infarct volume = sum of infarct volume of all sections measured (corrected infarct area × 2 mm for each section)/total contralateral hemispheric volume × 100.

### Neurological Score

After 24 h of reperfusion after MCAO, we conducted a modified neurological severity score (mNSS) to evaluate the neurological dysfunction, including reflexes absent & abnormal movements (score 0 to 2), beam balance tests (score 0 to 6), motor tests (including flexion of forelimb, flexion of hindlimb and head movement, score 0 to 6). Cumulative scores of 1 to 4, 5 to 9, or 10 to 14 revealed slight, moderate, or serious damage, respectively. Neurological performance was evaluated by independent blind researchers.

### Morris Water Maze (MWM) Test

In this study, the MWM test was used to examine the spatial learning and memory as we described earlier 29. In short, the water maze contained circular pool, which was 60 cm in height and 120 cm in diameter together with a 6- centimeter diametric circular platform. Before the test, the tank was filled with water supplemented with titanium oxide and ensure water submerges the platform 1 cm below the surface at 22 ± 2 °C. The tank was pasted with different shapes as spatial reference clues. Mice were placed in the operating room to apply to experimental environment for 24 hours before the test. The whole experiment lasted 7 days. Briefly, for the first 6 consecutive days, each of the groups was placed from the edge of each quadrant of the tank and allowed 60 s to search the hidden circular platform. Each group was received four training sessions. If the mouse did not look for the hidden platform within the specified time, boot it to the platform for 15 s. At 24 hours after the whole training, the probe test was performed. Before testing, taken out the platform, and recorded the animal's behavior for 60 s which contained the time spent in target quadrant, the latency of reaching the platform and the times of crossing the platform areas. The swimming track in the water maze was recorded by a digital tracking device (Xinruan Information Technology, Shanghai, China).

### Novel object recognition

Hippocampal-dependent memory was examined by novel object recognition tasks. First, the tested animals were placed in the test room to adapt the environment for 30 min on two consecutive days. At the first point of the test, the animals were placed in an open field device (50×50×50 cm) with two identical objects (familiar objects), placed in the left and right rear corners of the test box, and allowed free exploration of objects for 5 min. An hour later, replace a familiar object with a 55-milliliter glass bottle (a novel object) and place the animals in the arena again for a five-minute free-form exploration. Meanwhile, the observer recorded the time spent in exploring novel and familiar objects by the digital video tracking system (Xinyuan Information Technology). Facing the object (distance less than 2 cm) or making contact with an object (except the tail) was defined as an exploration. Index of discrimination was conducted as the ratio of the time spent contacting new objects to the total time spent contacting novel and familiar objects.

### Rotarod Test

Motor function was evaluated by a 6-cm-diameter rotating cylinder with rough surface. The mice were placed in a behavioral room to accustom the environment for 30 minutes. Next, the mice were given a 30 min training session and placed in the center and trained at a merry-go-round pace of 5 to 10 revolutions per minute (rpm). After that, the rotarod's speed increased from 5 rpm to 40 rpm in 5 min. The detector automatically records the time when each mouse falls from the rotating bar. Each experimental animal was repeated for four consecutive trials, each with a 30-minute break, and the data were statistically analyzed.

### Statistical analysis

Data were presented as the mean ± S.E.M. from at least three independent experiments. The statistical analyses were performed using the software GraphPad Prism software (version 8.0.1, GraphPad Software, Inc.). We performed the unpaired two-tailed Student's t-test for the experiments with only two groups. Multiple groups or more were compared by either one-way analysis of variance or two-way analysis of variance (ANOVA test). Significant effects in analysis of variances were followed by Dunnett's or Tukey's post hoc multiple comparison tests, as indicated in the figure legends. The discontinuous data of neurological deficits score and times of the animals crossing over the platform location during the probe trial on day 7 was assessed by Kruskal-Wallis non-parametric test, followed by Dunnett's post hoc test. *P* values less than 0.05 was defined as statistical significance.

## Results

### SENP6 mediated deSUMOylation of ANXA1 after cerebral ischemia-reperfusion injury

**Figure 1:**
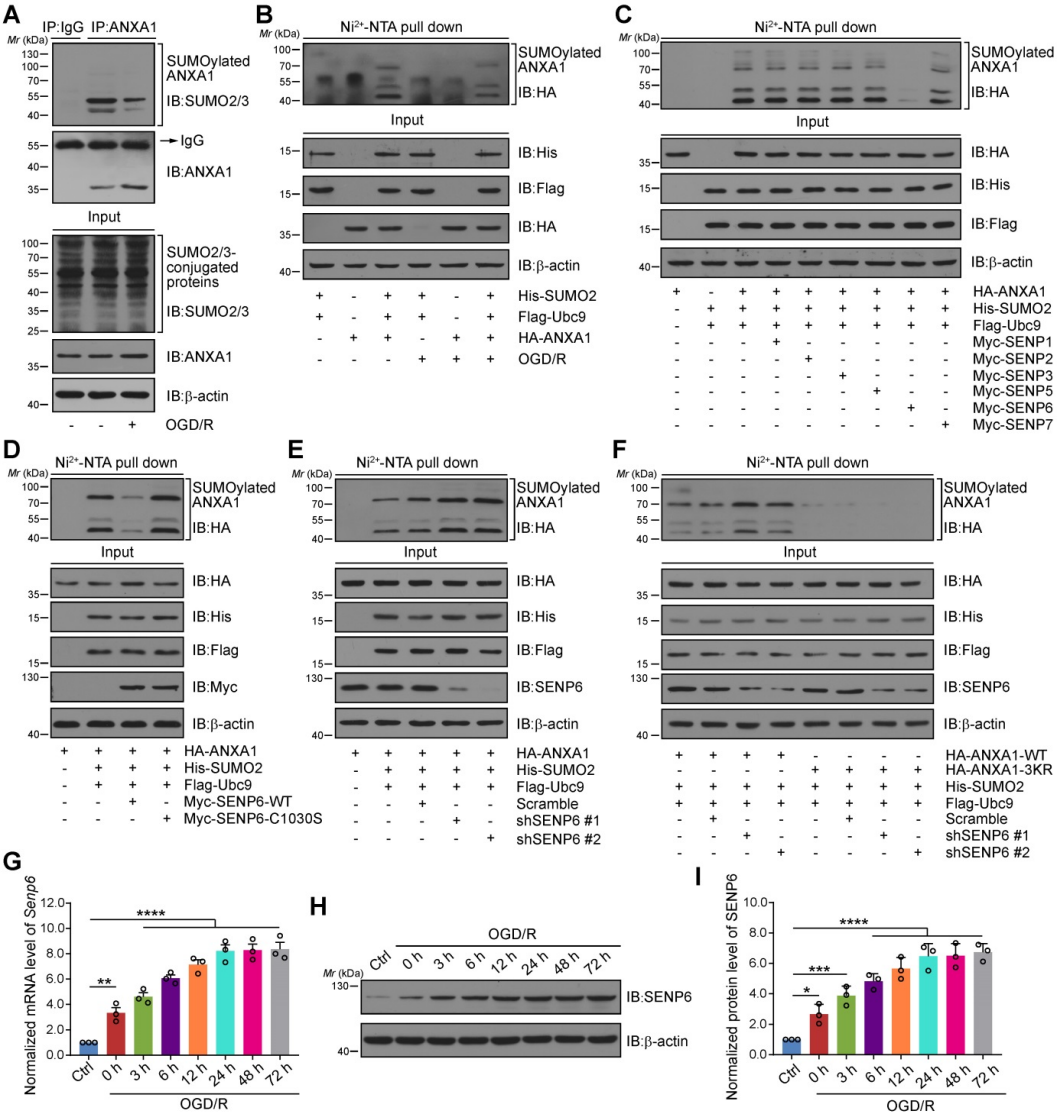
**SENP6 mediated the deSUMOylation of ANXA1.** (A) OGD/R inhibited the SUMOylation level of endogenous ANXA1. Primary cultured neurons were challenged with OGD/R, and whole cell lysates were used for IP assays for SUMOylation detection. (B) Representative Ni^2+^-NTA agarose affinity pull-down assay results showing the SUMOylation of ANXA1 in HEK293T cells treated with or without OGD/R. (C) SENP6 induced the deSUMOylation of ANXA1. HEK293T cells were transfected with Flag-Ubc9, His-SUMO2, HA-ANXA1 and different Myc-SENPs. A Ni^2+^-NTA affinity pull-down assay was used for SUMOylation detection. (D) Overexpression of wild-type SENP6, but not catalytic mutant C1030S, decreased the SUMOylation level of ANXA1. Flag-Ubc9, His-SUMO2, HA-ANXA1 with Myc-SENP6-WT or Myc-SENP6-C1030S were transfected into HEK293T cells, and the SUMOylation assay was performed with Ni^2+^-NTA agarose. (E) Silencing of SENP6 increased ANXA1 SUMO modification. SENP6 was knocked down by two specific shRNAs in HEK293T cells. Whole-cell lysates transduced with the indicated plasmids were subjected to Ni^2+^-NTA agarose. (F) Representative Ni^2+^-NTA agarose affinity pull-down assay results showing the SUMOylation of wild-type ANXA1 or ANXA1-3KR mutant in HEK293T cells treated with shRNA against SENP6. (G) OGD/R increased the *Senp6* mRNA level in neurons. qRT-PCR was conducted to examine the *Senp6* mRNA level. (H) OGD/R increased the protein levels of SENP6. Primary cultured neurons were treated with OGD and reoxygenation at the indicated time points. Whole cell lysates were subjected to immunoblots. (I) The quantitative analysis of SENP6 in (H). Data are reported as the mean ± S.E.M. from at least three independent experiments and analysed by one-way ANOVA followed by Dunnett's post hoc test. *P < 0.05, **P < 0.01, ***P < 0.001, ****P < 0.0001.

We first confirmed whether ANXA1 could be modified by SUMOylation in neurons after cerebral ischemia. Consistent with our previous study, we found that ANXA1 could be modified by SUMOylation and that this modification was greatly weakened in primary cultured neurons subjected to OGD/R (Figure 1A). Ni^2+^-NTA agarose affinity pull-down assay results also provided strong evidence that the SUMOylation level of ANXA1 was decreased after OGD/R challenge (Figure 1B). Next, we sought to explore which SENP enzyme mediated the deSUMOylation of ANXA1. HA-tagged ANXA1 together with a panel of Myc-tagged SENPs was transiently transfected into HEK293T cells. The results revealed that transfection of SENP6, rather than other members of the SENP family, decreased the SUMOylation of ANXA1 (Figure 1C). We next transfected a construct expressing wild-type (WT) or a catalytic mutant SENP6 into HEK293T cells. Compared to WT SENP6, overexpression of catalytic-domain-null SENP6-C1030S (Cysteine-to-Serine mutation at residue 1030 32, 33) lost the ability to deconjugate SUMO from ANXA1. Intriguingly, we found that SENP6-C1030S could enhance the SUMOylation of ANXA1, suggesting a dominant-negative loss-of-function effect of the catalytically dead mutant (Figure 1D). Meanwhile, affinity pull-down assay results revealed that SUMOylation of ANXA1 was increased when endogenous SENP6 was knocked down by using two specific short hairpin RNAs (shRNAs) in HEK293T cells (Figure 1E). Lysine 113, 161 and 257 (K113/161/257) are the major SUMO modification sites of ANXA1 16, and knockdown of SENP6 reversed the SUMOylation of WT ANXA1, but had little impact on the triple-mutant (K113/161/257R, 3KR) ANXA1 (Figure 1F). Our previous study reported that SUMOylation of ANXA1 also takes places in microglia, so we attempt to examine whether SENP6 response to deSUMOylation of ANXA1 in microglia. As shown in Figure S1, overexpression of SENP6 markedly decreased the SUMOylation level of ANXA1 in primary cultured microglia. Next, a time course study revealed that the mRNA levels of *Senp6* in primary cultured neurons gradually increased after OGD treatment (Figure 1G). Similarly, immunoblots also confirmed that the SENP6 protein level gradually increased after OGD/R treatment (Figure 1H-I). To exclude whether a temporal effect result in the increasing of SENP6 mRNA and protein expression, we found that there is no significant difference of SENP6 mRNA and protein level between different time point under normal conditions (Figure S2). Last, the SENP6 level was further investigated *in vivo*. Consistent with the *in vitro* data, qRT-PCR and immunoblots results also showed that the mRNA and protein level of SENP6 gradually increased after the onset of ischemic stroke (Figure S3). Collectively, these results conclusively confirm that cerebral ischemia induces the expression of SENP6 at both the RNA and protein levels and deconjugates SUMO2/3 chains from the ANXA1 protein.

### OGD/R enhanced the interaction of ANXA1 with SENP6

**Figure 2:**
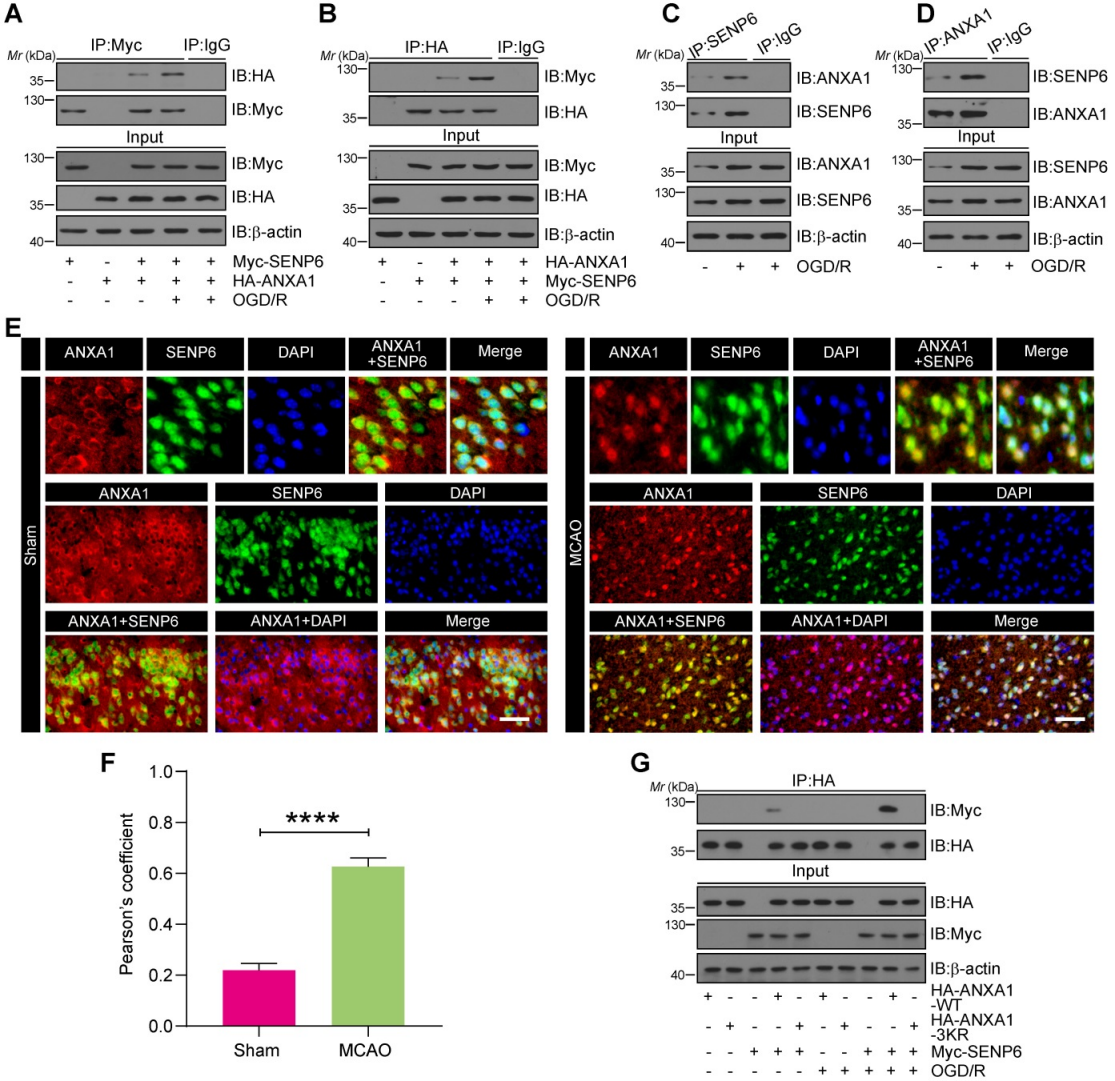
** OGD/R enhanced the interaction between SENP6 and ANXA1.** (A and B) Co-IP assay detecting exogenous SENP6-ANXA1 binding *in vitro*. HEK293T cells transiently expressing Myc-SENP6 and HA-ANXA1 were subjected to immunoprecipitation with an anti-Myc or HA antibody, followed by immunoblotting analysis with an anti-HA or Myc antibody. (C and D) Endogenous interaction of SENP6 and ANXA1. Primary cultured neurons were treated with OGD/R, and the cell lysates were subjected to immunoprecipitation with an anti-SENP6 or anti-ANXA1 antibody, followed by immunoblotting analysis with an anti-ANXA1 or anti-SENP6 antibody. (E) Representative immunofluorescence images showing the colocalization of ANXA1 and SENP6 in the cortex region of the mouse brain; the scale bar represents 40 µm. (F) Quantitative analysis of the colocalization was conducted by Pearson's coefficient measurement using ImageJ software. (G) SENP6 upregulation enhanced the interaction between ANXA1-WT and SENP6 but had no effect on the association of ANXA1-3KR with SENP6. HEK293T cells cotransfected with HA-ANXA1-WT or HA-ANXA1-3KR with or without Myc-SENP6 were subjected to immunoprecipitation with an anti-HA antibody followed by immunoblotting analysis with an anti-Myc antibody. All experiments are representative of three independent experiments. Data in panel (F) are presented as the mean ± S.E.M. and analysed by unpaired two-tailed Student's t-test. ****P < 0.0001.

To confirm whether ANXA1 is a putative SENP6 substrate, we first examined whether ANXA1 interacts with SENP6. Co-immunoprecipitation (Co-IP) assays showed that exogenous ANXA1 could bind with SENP6, and this interaction was greatly enhanced in HEK293T cells after OGD/R (Figure 2A). Reverse Co-IP assays also confirmed this result (Figure 2B). Meanwhile, we conducted an endogenous Co-IP assay of primary cultured neurons and showed that the interaction of endogenous ANXA1 with SENP6 was greatly increased after OGD/R (Figure 2C-D). In the input lanes of co-IP assays, we repeatedly observed that the ANXA1 protein level was increased after OGD/R treatment. These results were consistent with the fact that OGD/R could upregulate the expression of ANXA1, as reported by our previous study 14. Next, the interaction of ANXA1 with SENP6 were validated by immunofluorescence analysis, which showed the colocalization of endogenous SENP6 with ANXA1 in the cortex region of mice. As expected, the results also showed that the colocalization of endogenous SENP6 with ANXA1 in the cortex region of mice was significantly increased after cerebral ischemia (Figure 2E-F). Since SENP6 and ANXA1 could be detected both at cytosol and nucleus parts, co-IP experiments were performed to explore the interaction between SENP6 and ANAX1 in different cell fractionations. As shown in Figure S4, the results showed that the interaction of endogenous ANXA1 with SENP6 was greatly increased in both the cytoplasmic and nuclear fractionations after OGD/R treatment. Finally, Myc-tagged SENP6 together with HA-tagged ANXA1-WT or ANXA1-3KR was transiently transfected into HEK293T cells. We found that the interaction of ANXA1-WT and Myc-SENP6 increased after OGD/R, but no interaction between ANXA1-3KR and Myc-SENP6 was observed even upon OGD/R treatment (Figure 2G). Taken together, these results demonstrated that OGD/R enhanced the interaction of ANXA1 with SENP6.

### DeSUMOylation of ANXA1 by SENP6 promotes ANXA1 nuclear localization

**Figure 3:**
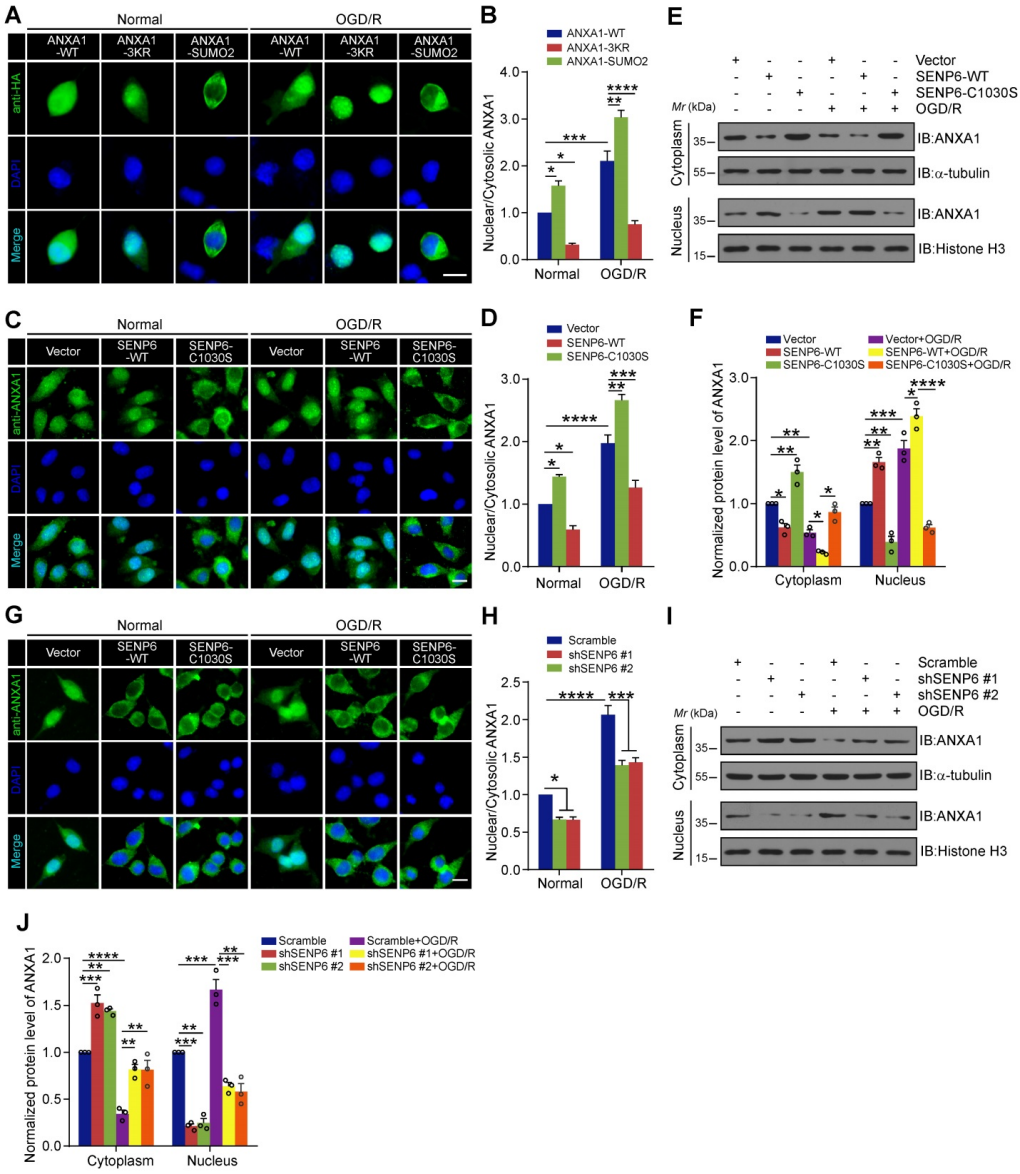
** SENP6 promotes nucleus translocation of ANXA1 after OGD/R.** (A) Representative immunofluorescence imaging showed the subcellular distribution of ANXA1 or its mutant in N2a cells infected with adenovirus encoding HA-tagged wild-type ANXA1, ANXA1-3KR or ANXA1-SUMO2. (B) Quantitative analysis of nuclear/cytoplasmic ANXA1 levels in (A). (C) Immunofluorescence imaging of stained ANXA1 showing the subcellular distribution of endogenous ANXA1. N2a neuronal cells were infected with adenovirus encoding wild-type or the catalytic mutant SENP6 and treated with or without OGD/R. (D) Quantitative analysis of nuclear/cytoplasmic ANXA1 levels in (C). (E) Immunoblots showing the relative amounts of ANXA1 in cytoplasmic and nuclear extracts of N2a cells infected with adenovirus encoding wild-type or the catalytic mutant SENP6 under normal or OGD/R conditions. (F) Quantitative analysis of the protein levels of ANXA1 in (E). (G) Immunofluorescence imaging of stained ANXA1 showing the subcellular distribution of endogenous ANXA1. N2a neuronal cells were infected with adenovirus encoding shRNAs against SENP6 or a nontargeting scramble shRNA and treated with or without OGD/R. (H) Quantitative analysis of nuclear/cytoplasmic ANXA1 levels in (G). (I) Immunoblots showing the relative amounts of ANXA1 in cytoplasmic and nuclear extracts of N2a cells infected with adenovirus encoding shRNAs against SENP6 under normal or OGD/R conditions. α-Tubulin and Histone H3 were used as cytoplasmic and nuclear loading controls, respectively. (J) Quantitative analysis of the protein levels of ANXA1. The intensity of the bands was quantitated by scanning densitometry and normalized to the values obtained for the control group. Scale bar = 10 µm. Data are presented as the mean ± S.E.M. from at least three independent experiments and analysed by two-way ANOVA followed by Tukey's post hoc test. *P < 0.05, **P < 0.01, ***P < 0.001 and ****P < 0.0001.

Because SUMOylation regulates the translocation of many proteins between the nucleus and cytoplasm 34, 35, we questioned whether SENP6-mediated deSUMOylation of ANXA1 regulates its subcellular localization. We first generated a SUMO2 fusion-directed SUMO modification system to efficiently and selectively investigate ANXA1 SUMOylation. To this end, SUMO2 was fused to the C-terminus of wild-type ANXA1 (a construct named ANXA1-SUMO2) as we previously reported 16. Next, we explored the functional significance of ANXA1 SUMOylation on subcellular localization. Immunofluorescence analysis showed that OGD/R increased the accumulation of WT ANXA1 in the nuclear fraction of N2a cells, whereas the ANXA1 3KR mutant primarily localized to the nucleus. In contrast to ANXA1-3KR, the ANXA1-SUMO2 construct exhibited cytoplasmic localization under normal and OGD/R conditions (Figure 3A-B). To further validate the effect of SENP6-mediated deSUMOylation of ANXA1 on its subcellular localization, adenoviruses encoding SENP6-WT and SENP6-C1030S were used to infect N2a cells, and we found that the nuclear translocation of ANXA1 obviously increased upon SENP6-WT transfection. More importantly, SENP6-C1030S overexpression markedly reversed the nuclear accumulation of ANXA1 and increased cytoplasmic ANXA1 after OGD/R (Figure 3C-D). These results were further confirmed by nuclear/cytosolic fractionation assays (Figure 3E-F). Finally, we knocked down SENP6 by specific shRNAs in N2a cells. Immunofluorescence assays demonstrated that shRNA-mediated SENP6 knockdown significantly decreased the nuclear accumulation of ANXA1 induced by OGD/R (Figure 3G-H). These results were also validated by nuclear/cytosolic fractionation assays (Figure 3I-J). Collectively, these data suggest that SENP6 functions as a positive regulator to induce ANXA1 nuclear translocation after OGD/R.

### SENP6-mediated deSUMOylation of ANXA1 enhances its serine phosphorylation

**Figure 4:**
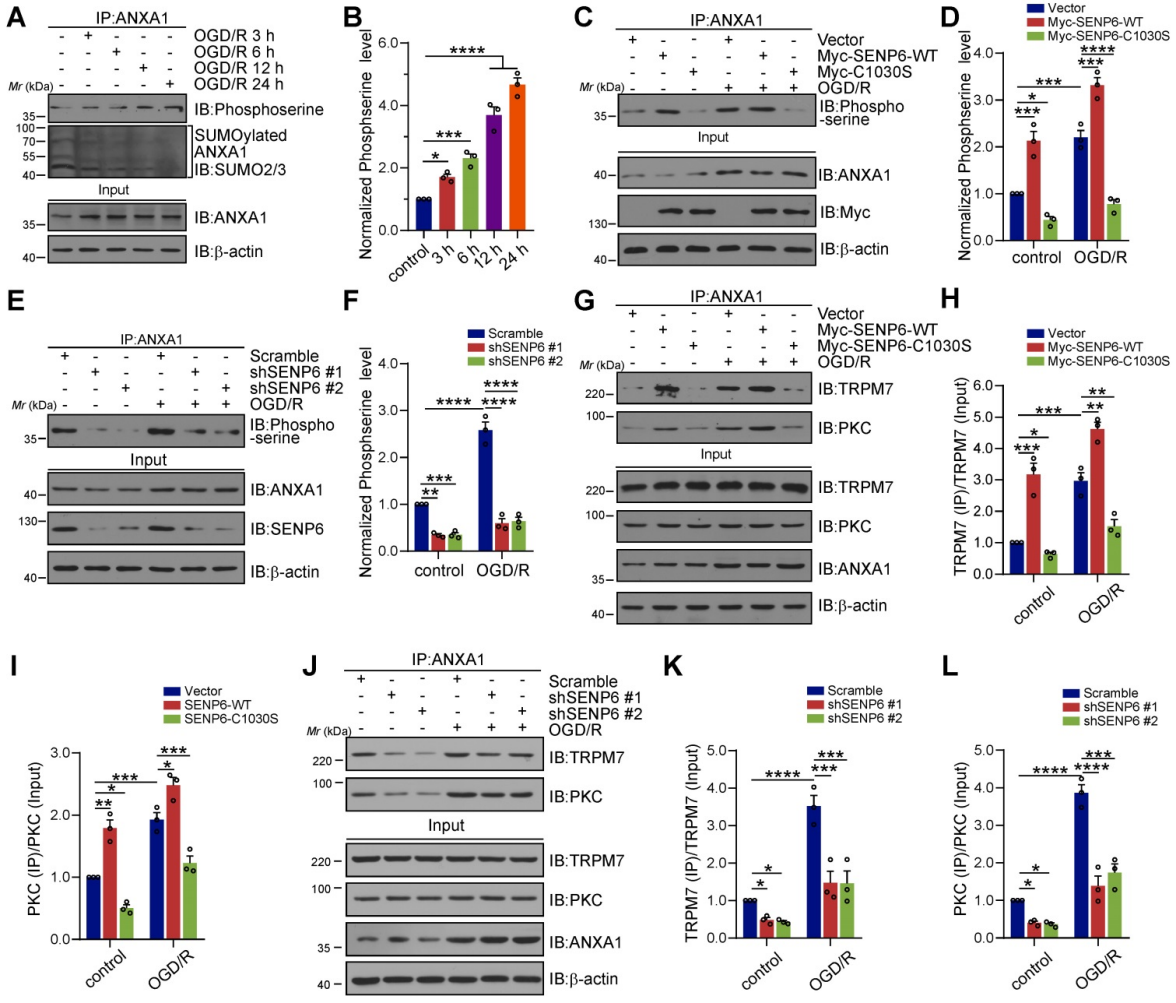
** SENP6-mediated deSUMOylation of ANXA1 enhances its serine phosphorylation.** (A) Primary cultured neurons were subjected to OGD for 1 h and then reperfusion for the indicated times. Co-IP assays confirmed the levels of ANXA1 SUMOylation and serine phosphorylation. (B) Quantification of the serine phosphorylation level of ANXA1. (C) Primary neurons were infected with adenovirus encoding SENP6-WT or SENP6-C1030S and were then subjected to OGD/R treatment or no treatment. Co-IP confirmed the levels of ANXA1 phosphorylation. (E) Primary neurons were infected with adenovirus encoding shRNAs against SENP6 or nontargeted scramble control and then subjected to OGD for 1 h and reperfusion for 24 h. Co-IP confirmed the levels of ANXA1 phosphorylation. (G) Primary neurons were infected with adenovirus encoding SENP6-WT or SENP6-C1030S and then subjected to OGD/R treatment or no treatment. Co-IP confirmed the interaction of endogenous ANXA1 and TRPM7 or PKC. (H and I) Quantification of the TRPM7 or PKC binding with ANXA1. (J) Primary neurons were infected with adenovirus encoding shRNAs against SENP6 or nontargeted scramble control, and then subjected to OGD/R treatment or no treatment. Co-IP confirmed the interaction of endogenous ANXA1 and TRPM7 or PKC. (K and L) Quantification of the TRPM7 or PKC binding with ANXA1. Statistical difference in panel (B) was determined one-way ANOVA followed by Dunnett's post hoc test, and all others were analysed by two-way ANOVA followed by Tukey's post hoc test. Data are presented as mean ± S.E.M. *P < 0.05, **P < 0.01, ***P < 0.001 and ****P < 0.0001.

Our previous study demonstrated that TRPM7 kinase-dependent phosphorylation of Ser^5^ and PKC kinase-dependent phosphorylation of Ser^27^ are critical for ANXA1 nuclear translocation 13, 36. To understand the mechanisms underlying SENP6-induced ANXA1 nuclear shuttling, we further explored the interplay between ANXA1 phosphorylation and SUMOylation. A time course study revealed that the SUMOylation levels of ANXA1 in primary cultured neurons gradually decreased after OGD/R, while the phosphorylation level of ANXA1 showed the opposite effect. ANXA1 serine phosphorylation gradually increased after the onset of OGD/R (Figure 4A-B). Next, adenoviruses encoding SENP6-WT and SENP6-C1030S were used to infect primary cultured neurons. The immunoblot results showed that OGD/R greatly induced the phosphorylation of ANXA1, and this effect was further enhanced by SENP6-WT overexpression. In contrast, treatment with SENP6-C1030S caused a substantial decrease in ANXA1 serine phosphorylation (Figure 4C-D). In addition, we found that knockdown of SENP6 by two independent shRNAs in primary neurons significantly decreased the phosphoserine level of ANXA1 under normal and OGD/R conditions (Figure 4E-F). Co-IP experiments also demonstrated that SENP6-WT markedly enhanced the interaction of ANXA1 with TRPM7 and PKC kinases. However, SENP6-C1030S exhibited the opposite effect and significantly decreased the interaction between ANXA1 and TRPM7 and PKC kinases in primary cultured neurons under normal or OGD/R conditions (Figure 4G-I). Last, shRNAs-mediated SENP6 knockdown significantly decreased the interaction of ANXA1 with TRPM7 and PKC kinases (Figure 4J-L). Interestingly, as shown in the input lanes in Figure 4G and 4J, overexpression of SENP6-WT or SENP6-C1030S, and shRNAs-mediated SENP6 knockdown had little impact on the protein level of TRPM7 and PKC kinases in primary cultured neurons. In addition, lentivirus-mediated overexpression of SENP6-WT or SENP6-C1030S had little impact on the protein level of TRPM7 and PKC in mice subjected to Sham or MCAO operation (Figure S5). These results demonstrated that SENP6 doesn't regulate the levels of TRPM7 and PKC kinases. Taken together, the above data indicated that SENP6 could enhance ANXA1 phosphorylation and that this effect depended on its deSUMOylation enzyme activity.

### Inhibition of SENP6 attenuates OGD/R-induced p53 transcriptional activity, Bid expression and caspase-3 apoptosis pathway activation

**Figure 5:**
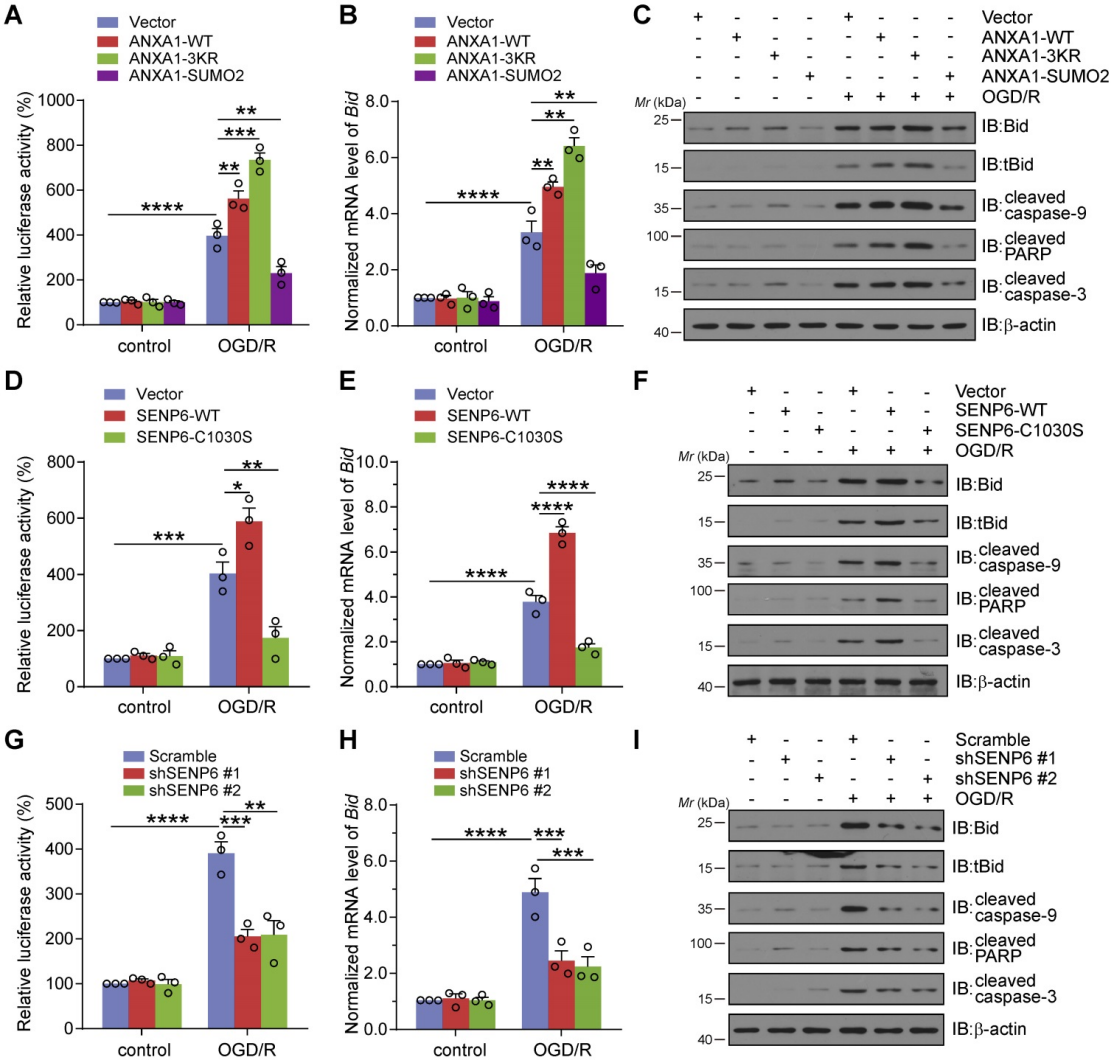
**Inhibition of SENP6 attenuates OGD/R-induced p53 transcriptional activity, Bid expression and caspase-3 apoptosis pathway activation.** (A) Luciferase reporter assay results showed the transcriptional activity of p53 in HEK293T cells transfected with wild-type ANXA1, ANXA1-3KR or ANXA1-SUMO2. (B) qRT-PCR analysis of *Bid* mRNA expression following overexpression of wild-type or different mutants of ANXA1 in primary neurons. (C) Representative immunoblots showing the protein levels of tBid, cleaved caspase-9, cleaved PARP, and cleaved caspase-3 in primary neurons infected with adenovirus encoding wild-type ANXA1, ANXA1-3KR or ANXA1-SUMO2. (D) Luciferase report assay results showed the transcriptional activity of p53 in HEK293T cells transfected with SENP6-WT or SENP6-C1030S. (E) qRT-PCR analysis of *Bid* mRNA expression following SENP6-WT or SENP6-C1030S overexpression in primary neurons treated with or without OGD/R. (F) Representative immunoblots showing the protein levels of tBid, cleaved caspase-9, cleaved PARP, and cleaved caspase-3 in primary neurons infected with adenovirus encoding SENP6-WT or SENP6-C1030S. (G) Luciferase report assay results showed the transcriptional activity of p53 in HEK293T cells transfected with shRNA against SENP6 or scramble control. (H) qRT-PCR analysis of *Bid* mRNA expression in primary neurons following SENP6 shRNA or scramble control infected under normal or OGD/R conditions. (I) Representative immunoblots showing the protein levels of tBid, cleaved caspase-9, cleaved PARP, and cleaved caspase-3 in primary neurons infected with adenovirus encoding SENP6 shRNA or scramble control. Data are presented as the mean ± S.E.M. from at least three independent experiments and analysed by two-way ANOVA followed by Tukey's post hoc test. *P < 0.05, **P < 0.01, ***P < 0.001 and ****P < 0.0001.

Our previous studies suggested that cerebral ischemia-induced ANXA1 nuclear translocation enhanced p53 transcriptional activity, increased proapoptotic Bid expression and subsequently activated the caspase-3 proapoptotic pathway, eventually leading to neuronal apoptosis 14. We then conducted experiments to explore the effect of SENP6-mediated deSUMOylation of ANXA1 on neuronal damage after cerebral ischemia. Therefore, we first employed a dual luciferase reporter assay to measure p53 transcriptional activity. The results suggested that OGD/R increased p53 transcriptional activation, as expected. In addition, compared with the vector control, ANXA1-WT overexpression resulted in increased p53 transcriptional activity. More importantly, ANXA1-3KR overexpression led to further increased p53 transcriptional activity. However, p53 transcriptional activity was reversed by ANXA1-SUMO2 overexpression (Figure 5A). Next, adenoviruses encoding WT ANXA1, ANXA1-3KR, and ANXA1-SUMO2 were used to infect primary cultured neurons to explore the different SUMOylation states of ANXA1 on Bid expression and caspase-3 apoptosis pathway activation. qRT-PCR and immunoblot results indicated that ANXA1-3KR significantly increased Bid mRNA and protein levels. In addition, ANXA1-3KR also significantly increased OGD/R-triggered truncated Bid (tBid) expression and caspase-9, poly-ADP-ribose polymerase (PARP), and caspase-3 cleavage. Conversely, ANXA1-SUMO2 overexpression presented exactly the opposite effect (Figure 5B-C). Next, a dual luciferase reporter assay was used to examine the effect of SENP6 on p53 transcriptional activity. The results revealed that SENP6-WT greatly increased p53 transcriptional activity, while SENP6-C1030S reversed OGD/R-induced transcriptional activation of p53 (Figure 5D). Furthermore, adenoviruses encoding SENP6-WT and SENP6-C1030S were used to infect primary neurons, and qRT-PCR and immunoblots showed that SENP6-C1030S significantly inhibited OGD/R-induced Bid expression and caspase-9, PARP, and caspase-3 cleavage (Figure 5E-F). Finally, knockdown of SENP6 strongly restricted OGD/R-induced p53 transcriptional activity and Bid expression and caspase-9, PARP, and caspase-3 cleavage (Figure 5G-I). Taken together, these data collectively suggest that inhibition of SENP6 restricts OGD/R-induced p53 transcriptional activity, Bid expression and caspase-3 apoptosis pathway activation.

In these experimental results, we repeatedly observed that the overexpression or knockdown of SENP6 affect the SUMOylation and phosphorylation of ANXA1 but with no obvious effects on the transcriptional activity of p53 and transcriptional level of Bid at normal condition. These results prompted us to explore the underlying mechanism. So, we next undertook to examine the effect of SENP6 overexpression or knockdown on the binding of ANXA1 with p53. As shown in Figure S6, primary cultured neurons were infected with adenoviruses expressing Vector, SENP6-WT, SENP6-C1030S, shRNA against SENP6 or scrambled control, respectively. The co-IP results showed that the binding of ANXA1 with p53 was strongly increased after OGD/R treatment. Interestingly, overexpression of SENP6-WT enhanced the interaction of ANXA1 with p53, while shRNA mediated SENP6 knockdown inhibited this interaction between ANXA1 and p53 only under OGD/R conditions. In contrast, the interaction between ANXA1 and p53 was very weak in the normal conditions, and overexpression or knockdown of SENP6 had little effect on this interaction.

Therefore, under normal conditions, the overexpression or knockdown of SENP6 has little effect on the transcriptional activity of p53 and the transcriptional level of Bid, which may be due to the weak interaction between ANXA1 and p53.

### Inhibition of SENP6 alleviates neuronal apoptosis after OGD/R

**Figure 6:**
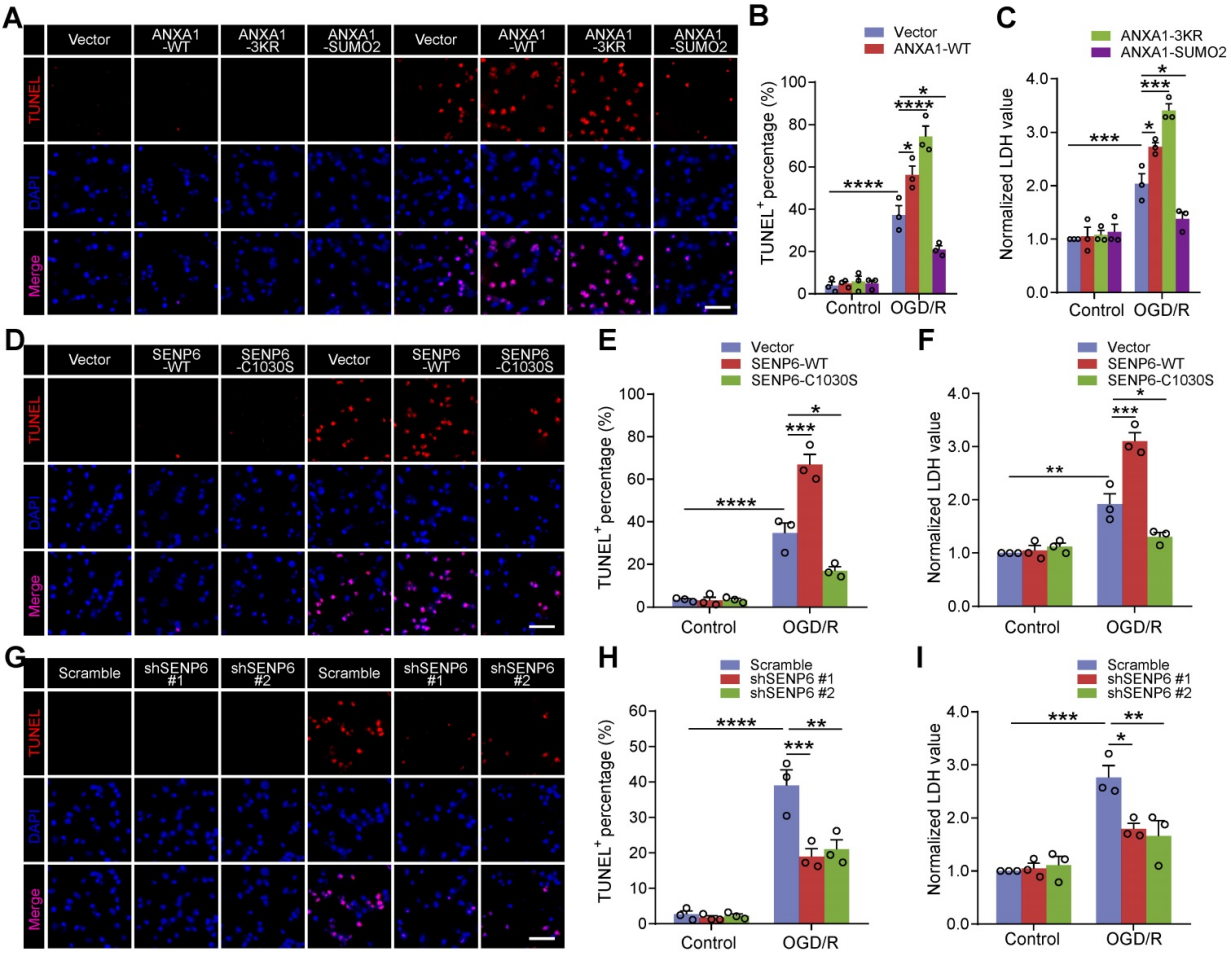
** Inhibition of SENP6 restrains neuronal cell apoptosis induced by OGD/R.** (A and B) Representative TUNEL staining and quantitative analysis showing the effect of wild-type or mutant ANXA1 overexpression on neuronal apoptosis after OGD/R. Primary cultured neurons were infected with adenoviral vectors carrying wild-type or mutant ANXA1 and were then treated with OGD/R. (C) LDH assay indicating the effect of wild-type or mutant ANXA1 overexpression on LDH release in primary cultured neurons under normal and OGD/R conditions. (D and E) Primary cultured neurons were infected with adenovirus encoding SENP6-WT or SENP6-C1030S and were then subjected to OGD/R treatment or no treatment. Representative TUNEL staining and quantitative analysis were conducted to examine cell apoptosis. (F) LDH assay indicating the effect of wild-type or catalytic mutant SENP6 overexpression on LDH release in primary cultured neurons under normal and OGD/R conditions. (G and H) Primary cultured neurons were infected with adenovirus encoding shRNAs against SENP6 or a nontargeting scramble shRNA and were then subjected to OGD/R treatment or no treatment. Representative TUNEL staining and quantitative analysis was conducted to examine the cell apoptosis. (I) The LDH assay indicating the effect of shRNA-mediated SENP6 deficient on LDH release in primary cultured neurons under normal and OGD/R conditions. Scale bar = 40 µm. Data are presented as the mean ± S.E.M. from at least three independent experiments and analysed by two-way ANOVA followed by Tukey's post hoc test. *P < 0.05, **P < 0.01, ***P < 0.001 and ****P < 0.0001.

We next investigated the effect of SENP6 inhibition on neuronal apoptosis. TdT-mediated dUTP-X nick end labeling (TUNEL) staining revealed that OGD/R-triggered neuronal apoptosis was aggravated by ANXA1-WT overexpression, consistent with our previous report 14. Furthermore, we found that the ANXA1-3KR mutant exacerbated neuronal apoptotic death, while ANXA1-SUMO2 exhibited a profound protective effect against neuronal damage induced by OGD/R (Figure 6A-B). The lactate dehydrogenase (LDH) release assay also indicated that ANXA1-WT overexpression increased OGD/R-induced LDH release. More importantly, ANXA1-3KR overexpression further promoted OGD/R-induced LDH release, whereas OGD/R-induced LDH release was reversed by ANXA1-SUMO2 (Figure 6C). Furthermore, TUNEL staining also revealed that SENP6-C1030S overexpression inhibited OGD/R-induced neuronal damage (Figure 6D-E), and these results were also validated by LDH release induced by OGD/R (Figure 6F). Next, we infected primary neurons with the adenovirus vector carrying shRNA against SENP6 or scrambled control. As expected, TUNEL staining assays revealed that SENP6 shRNA alleviated neuronal apoptosis induced by OGD/R (Figure 6G-H). Consistently, SENP6 shRNA also significantly inhibited OGD/R-induced LDH release (Figure 6I). Taken together, these data strongly indicate that inhibition of SENP6 alleviates neuronal apoptosis after OGD/R.

### OGD/R-induced SENP6-dependent neuronal cell death is mediated by its deSUMOylation of ANXA1

**Figure 7:**
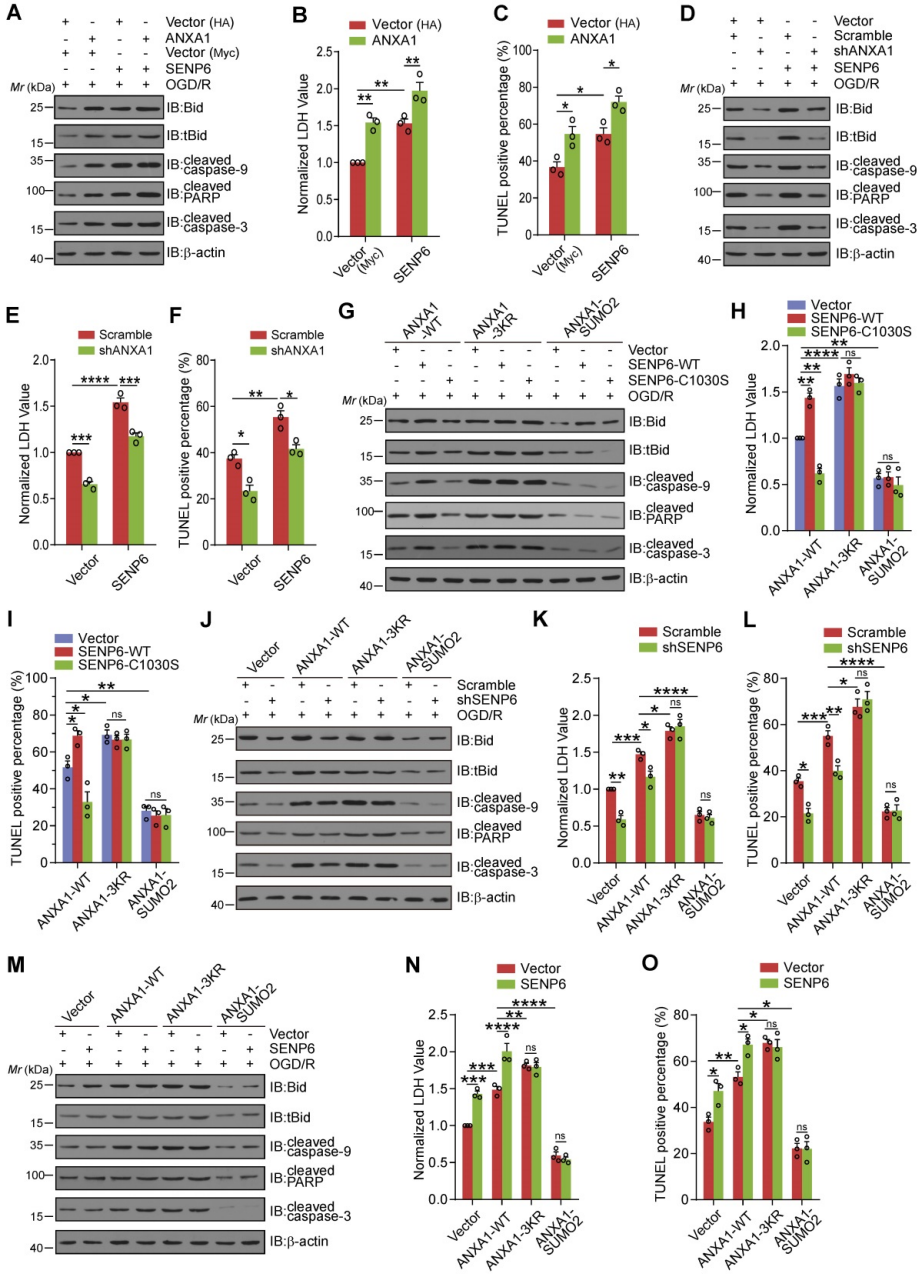
** SENP6 activated the caspase-3 pathway and induced neuronal apoptosis induced by OGD/R depending on its deSUMOylation of ANXA1.** (A) Representative immunoblots showing the protein levels of apoptosis-related proteins in primary neurons infected with ANXA1 or SENP6 alone or together. (B) LDH assay indicating the effect of ANXA1 and SENP6 overexpression alone or together on LDH release in primary neurons. (C) Quantitative analysis of TUNEL staining showing the effect of ANXA1 and SENP6 overexpression alone or together on neuronal apoptosis. (D) Representative immunoblots showing the protein levels of apoptosis-related proteins in primary neurons infected with ANXA1 shRNA or SENP6 alone or together. (E) LDH assay indicating the effect of ANXA1 shRNA or SENP6 overexpression alone or together on LDH release in primary neurons. (F) Quantitative analysis of TUNEL staining showing the effect of ANXA1 shRNA or SENP6 overexpression alone or together on neuronal apoptosis. (G) Representative immunoblots showing the protein levels of apoptosis-related proteins in primary neurons infected with wild-type or mutant ANXA1 together with SENP6-WT or SENP6-C1030S. (H) LDH assay indicating the effect of SENP6-WT or SENP6-C1030S upregulation in ANXA1-WT- or ANXA1-mutant-overexpressing neurons on LDH release in primary neurons under OGD/R conditions. (I) Quantitative analysis of TUNEL staining showing the effect of SENP6-WT or SENP6-C1030S upregulation in ANXA1-WT or mutant overexpression neurons on neuronal apoptosis after OGD/R. (J) Primary neurons were infected with adenoviral vectors carrying wild-type or mutant ANXA1 and SENP6 shRNA or a scramble control. Immunoblotting was conducted to examine the protein levels of apoptosis-related proteins. (K) LDH assay indicating the effect of wild-type or mutant ANXA1 overexpression with SENP6 silencing on LDH release in primary neurons. (L) Quantitative analysis of TUNEL staining showing the effect of wild-type or mutant ANXA1 overexpression with SENP6 silencing on neuronal apoptosis. (M) Primary neurons were infected with adenoviral vectors carrying wild-type or mutant ANXA1 and SENP6. Immunoblotting was conducted to examine the protein levels of apoptosis-related proteins. (N) LDH assay indicating the effect of wild-type or mutant ANXA1 with SENP6 overexpression on LDH release in primary neurons. (O) Quantitative analysis of TUNEL staining showing the effect of wild-type or mutant ANXA1 with SENP6 overexpression on neuronal apoptosis. Data are presented as the mean ± S.E.M. from at least three independent experiments and analysed by two-way ANOVA followed by Tukey's post hoc test. ns, no significance. *P < 0.05, **P < 0.01, ***P < 0.001, ****P < 0.0001.

Next, we explored whether SENP6 mediated neuronal apoptosis depended on its enzymatic activity in the deSUMOylation of ANXA1. We first examined SENP6-mediated cell death by ANXA1 overexpression or knockdown. We found that when primary cultured neurons were transduced with both SENP6 and ANXA1, Bid expression, caspase-9, PARP, and caspase-3 cleavage, LDH release and neuronal cell apoptosis were increased compared with cells expressing SENP6 or ANXA1 alone (Figure 7A-C). In addition, shRNA-mediated endogenous ANXA1 ablation significantly reversed SENP6-aggravated neuronal damage after OGD/R, suggesting that SENP6-dependent cell death may be mediated by ANXA1 (Figure 7D-F). Next, adenoviral-mediated SENP6-WT overexpression significantly increased Bid expression, caspase-9, PARP, and caspase-3 cleavage, LDH release and neuronal cell apoptosis. SENP6-C1030S showed a protective effect in WT ANXA1-overexpressing cells but not ANXA1-3KR- or ANXA1-SUMO2-overexpressing cells (Figure 7G-I). We then efficiently knocked down endogenous SENP6 expression using adenoviral-mediated shRNA. In contrast to the scramble control cells, when SENP6 expression was suppressed, Bid expression, caspase-9, PARP, and caspase-3 cleavage, LDH release and neuronal cell apoptosis were significantly decreased in WT ANXA1-overexpressing cells but had little impact on ANXA1-3KR- or ANXA1-SUMO2-overexpressing cells (Figure 7J-L). Finally, we found that adenoviral-mediated SENP6 overexpression in primary cultured neurons resulted in substantially increased Bid expression, caspase-9, PARP, and caspase-3 cleavage, LDH release and neuronal cell apoptosis in WT ANXA1-overexpressing cells but not in ANXA1-3KR- or ANXA1-SUMO2-overexpressing cells (Figure 7M-O). These data collectively indicate that SENP6-dependent neuronal cell death may depend on its enzymatic activity to mediate the deSUMOylation of ANXA1.

### Inhibition of SENP6 protects against cerebral ischemia-reperfusion injury *in vivo*

**Figure 8:**
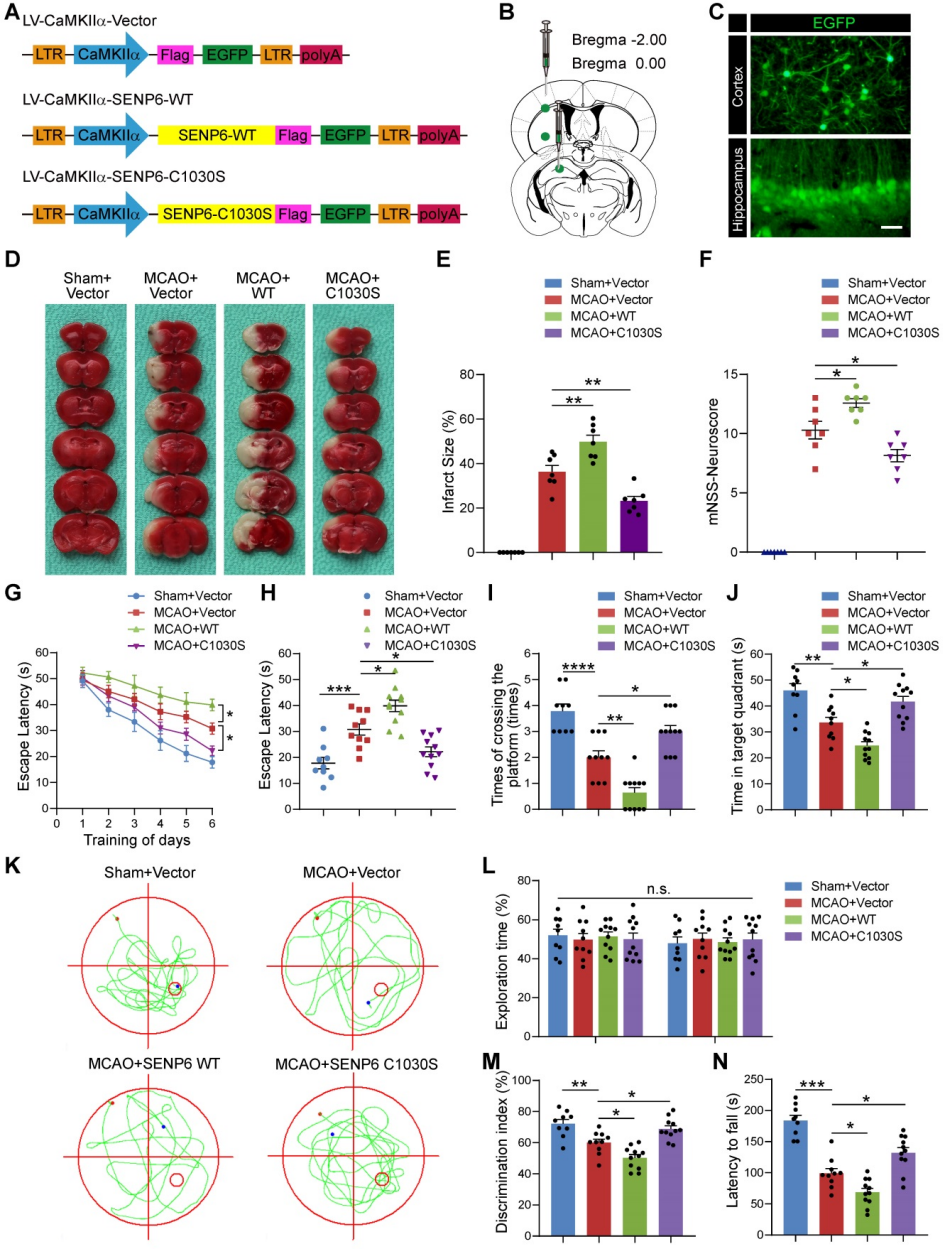
** Inhibition of SENP6 in neurons protects against cerebral ischemic-reperfusion injury in mice.** (A) Schematic of CamKⅡa-dependent lentiviruses vectors for neuron wild-type or catalytic mutant SENP6 overexpression. (B) Lentiviruses were injected into the hippocampus CA1 region, cerebral cortex, and striatum of adult male mice. (C) Representative images of EGFP signals of mice injected with the lentiviruses two weeks after virus injection. Scale bar = 20 µm. (D) Representative images of TTC staining indicated the infarct volume in each treatment group. n = 7 mice per group. (E) Quantitative analysis of infarct size. (F) Neurological scores of each group 72 h after reperfusion following MCAO. n = 7 mice per group. (G to K) Latency trial (G, H) and probe trial (I to K) results in the MWM tests. n = 9-11 mice per group. (G) Escape latency to reach the hidden platform during days 1-6 of testing. (H) Exploration time (in seconds, s) spent in searching of the hidden platform on day 6. (I) Number of times the mice crossed the target platform location during the probe trials on day 7. (J) Time (in seconds, s) spent in the target quadrant during the probe trials on day 7. (K) Representative swimming traces indicating the sample paths from the probe trials on day 7. (L, M) Preference index for the novel object in the novel object recognition (NOR) task. n = 9-11 mice per group. (L) Exploration time of the mice in the familiarization phase, and (M) discrimination index in the test phase. (N) The mean latency to fall off the rotarod drum during the final test. n = 9-11 mice per group. Statistical difference in panel (G) was determined by the RM ANOVA followed by Tukey's post hoc test. Data in panel (F and I) was assessed by Kruskal-Wallis non-parametric test, followed by Dunnett's post hoc test. Data in panel (L) were analysed by two-way ANOVA followed by Tukey's post hoc test, and all others were used one-way ANOVA followed by Dunnett's post hoc test. Numbers in bars, numbers of mice. Data are presented as mean ± S.E.M. n.s. for *P* > 0.05, *P < 0.05, **P < 0.01, ***P < 0.001 and ****P < 0.0001.

Our *in vitro* studies suggested that inhibition of SENP6-mediated deSUMOylation of ANXA1 could alleviate OGD/R-induced neuronal apoptotic death. We then sought to investigate its protective effects against cerebral ischemia-reperfusion injury *in vivo*. We employed a lentivirus-mediated overexpression approach to target neurons in the brains of mice. We constructed two neuron-specific Flag-SENP6-WT- and Flag-SENP6-C1030S-expressing lentiviral plasmids in which the expression of Flag-tagged SENP6-WT or SENP6-C1030S is under the control of the mouse CamKⅡa promoter 37. The same lentiviral vector expressing EGFP was used as an experimental control (Figure 8A). The viruses were stereotactically injected into the hippocampal CA1 region, cerebral cortex, and striatum of adult C57/BL6 male mice (Figure 8B). As verified, EGFP expression was observed in the brains of lentivirus-injected mice (Figure 8C). Two weeks after lentivirus injection, animals underwent the middle cerebral artery occlusion (MCAO) surgery for 1 h followed by reperfusion to establish a transient focal brain ischemia-reperfusion injury animal model. First, we detected the infarct volume via 2,3,5-triphenyltetrazolium chloride (TTC) staining 24 h after reperfusion. The results revealed a marked increase in the infarct volume in SENP6-WT-overexpressing mice but a great reduction in SENP6-C1030S-overexpressing mice compared with vector control mice (Figure 8D-E). Furthermore, we used the modified neurological severity score (mNSS) to evaluate neurological deficits and found that the scores of SENP6 C1030S-overexpressing animals were substantially lower than those of vector control mice (Figure 8F). To confirm whether the lentiviral treatment actually reduced neuronal apoptosis, we next performed experiments to measure TUNEL positive cells in the ischemic region. As shown in Figure S7, 1-h MCAO followed by 24-h reperfusion triggered a profound reduction in the number of surviving cells and an increase in the number of apoptotic cells in the hippocampus and cerebral cortex. In addition, SENP6-WT treatment significantly exacerbated neuron apoptosis, whereas treatment with SENP6-C1030S was profoundly neuroprotective. These data demonstrated that the lentiviral treatment with SENP6-C1030S actually reduced neuronal apoptosis.

We next utilized the Morris water maze (MWM) test to examine the spatial learning and memory function of the mice after MCAO. The results showed that SENP6-WT treatment significantly exacerbated evident cognitive dysfunction compared to the vector control. Conversely, SENP6-C1030S-overexpressing mice exhibited remarkable cognitive improvement (Figure 8G-K). Moreover, a novel object recognition task was performed to confirm the effect on the cognitive function following focal ischemic injury. The data demonstrated that SENP6-C1030S-treated mice remarkably exhibited a better preference for the novel object, suggesting that SENP6-C1030S-treated animals preserved a better memory of the familiar object (Figure 8L-M). Finally, we conducted the rotarod test to further research motor function. The results demonstrated that, as expected, SENP6-C1030S-treated animals displayed stable motor function and spent more time on the rotarod than the vector control animals (Figure 8N). Taken together, these data suggested that SENP6 inhibition by overexpression of a SENP6 enzyme-deficient mutant significantly reduced brain ischemic infarct size, diminished neurological deficit scores and preserved cognitive and motor function after cerebral ischemia stroke.

## Discussion

In this study, we reported and analyzed the effect of cerebral ischemia-induced deSUMOylation of ANXA1 on neuronal apoptosis and the underlying mechanism. Specifically, we found that SENP6 functions as a SUMO-specific isopeptidase to deconjugate SUMO from ANXA1. Interestingly, cerebral ischemia enhanced SENP6 expression at both the RNA and protein levels in neurons and enhanced the binding of SENP6 with ANXA1 *in vitro* and *ex vivo*. Moreover, we found that SENP6-mediated deSUMOylation of ANXA1 promotes nuclear transport. Based on this observation, we then investigated the underlying mechanism and found that deconjugation of SUMO from ANXA1 can enhance its serine phosphorylation. Further work revealed that deSUMOylation-induced ANXA1 nuclear transport could activate p53 transcriptional activity and the caspase-3 pathway, eventually resulting in neuronal apoptotic death after ischemic stroke. More importantly, this study also showed that SENP6 inhibition ameliorated the neurological functional outcomes of animals subjected to cerebral ischemic injury.

Growing evidence has implicated the importance of SUMOylation in the regulation of protein activity, stability, cellular distribution and interactions with other proteins 38-40. ANXA1 has previously been reported as a substrate for SUMO modification 41. Hirata *et al*. demonstrated that ANXA1 could be modified by SUMOylation and that this modification is important for its helicase activity under DNA damage 42. In a previously study, we revealed that ANXA1 was mainly conjugated to SUMO2/3 and that SUMOylation of ANXA1 was greatly decreased after cerebral ischemia 16. However, whether and how the deSUMOylation of ANXA1 contributes to neuronal damage in the brain in response to ischemic stroke injury remains unclear. Here, we showed that SENP6 functions as a specific isopeptidase of ANXA1 and that SENP6 could aggravate neuronal damage after cerebral ischemia. In light of this discovery, we further found that inhibition of SENP6, particularly in neurons, can alleviate neuronal apoptosis and protect against ischemic stroke. As the HEK293 cell line is suitable for transfection with exogenous gene expression plasmids or shRNA plasmids, we conducted most of the experiments in Figure 1 in HEK293T cells, thus we should notice the possible differences between HEK293T cells and neurons. Previous data showed that SUMOylated ANXA1 upregulation promoted the anti-inflammatory polarization of microglia subjected to OGD/R injury and that overexpression of SUMOylated ANXA1 specifically in microglia showed robust neuroprotection against ischemic stroke. As SUMOylated ANXA1 protects against ischemic stroke through different mechanisms in neurons and microglia, we hypothesized that increased SUMOylation of ANXA1 in neurons and microglia at the same time would be even more potent. Certainly, more detailed work will be needed to verify this hypothesis.

As shown in previous studies, ANXA1 performs different biological roles depending on its subcellular localization 43. ANXA1 membrane migration and secretion are involved in anti-inflammatory effects and neuronal survival during OGD/R 44-46. Cumulative evidence also suggests a role of ANXA1 nuclear translocation in neuronal apoptosis following ischemic stroke 13, 14, 29. Our previous data showed that TRPM7 kinase-phosphorylated ANXA1 at Ser^5^ and PKC kinase-phosphorylated ANXA1 at Ser^27^ are critical for ANXA1 nuclear translocation 13, 36. In the present study, we further found that the SUMOylation status of ANXA1 controls its subcellular localization. Indeed, SENP6-mediated deSUMOylation of ANXA1 can enhance its serine phosphorylation. Protein function is tightly regulated by reversible posttranslational modifications to create an on/off state that is crucial for many biological processes. Many proteins are dynamically modified at multiple sites by different modifications 47. The interplay between phosphorylation and SUMOylation has been shown to play an important role in regulating the activity of substrate proteins 48-51. For example, Van Nguyen *et al.* reported that SUMOylation of STAT5 inhibits its phosphorylation and subsequent signaling 52. Hietakangas* et al*. also confirmed that phosphorylation of Ser^303^ is a prerequisite for the stress-inducible SUMO modification of HSF1 53. In this study, we showed that ANXA1 deSUMOylation can induce its serine phosphorylation, which is probably correlated with TRPM7 and PKC kinases.

The SUMO protease SENP6 is an enzyme involved in the proteolytic removal of SUMO chains from target proteins and has a preference for SUMO2/3-modified substrates 54, 55. SENP6 plays an important role in the regulation of genome stability, cell division, autoimmune responses, adult hematopoietic stem cell renewal and cellular senescence 56-61, but whether and how SENP6 plays a role in cerebral ischemia remains to be investigated. In the present study, we demonstrated that SENP6 could deconjugate SUMO2/3 chains from ANXA1. These data are in keeping with the general view that SENP6 preferentially targets poly-SUMO chains 62. We showed that SENP6 physically interacted with ANXA1 to remove SUMO from the ANXA1 protein in normal and OGD/R conditions. Although deSUMOylation of ANXA1 by SENP6 had little effects on the transcriptional activity of p53 and transcriptional level of Bid at normal condition, these processes may have a critical role in maintaining a balance between the level of ANXA1 SUMOylation in the uninjured conditions, as the progress of SUMOylation is generally dynamic and reversable 23. In addition, overexpression of WT SENP6, but not the SENP6 mutant, reduced the ANXA1 SUMOylation level, facilitated its nuclear and eventually led to neuronal apoptotic death after cerebral ischemia. Thus, overexpression of WT SENP6 can exacerbate neuronal damage in mice subjected to ischemic stroke injury. In contrast, inhibition of SENP6 by overexpression of a SENP6 catalytic mutant or shRNAs significantly decreased neuronal cell death, resulting in robust neuroprotection against cerebral ischemic-reperfusion injury. Given the critical role of SUMOylated ANXA1 in microglial polarization and the inflammatory response after cerebral ischemia, SENP6 may also play a role in these processes. Indeed, the deSUMOylation of NLRP3 by SENP6 controls NLRP3 inflammasome activation in BMDMs 63. Liu *et al.* also demonstrated that SENP6 could regulate TLR-induced inflammatory signaling via the deSUMOylation of NEMO 32. Whether SENP6-mediated ANXA1 deSUMOylation has a role in microglial polarization and the inflammatory response after ischemic stroke warrants future investigation.

**Figure 9:**
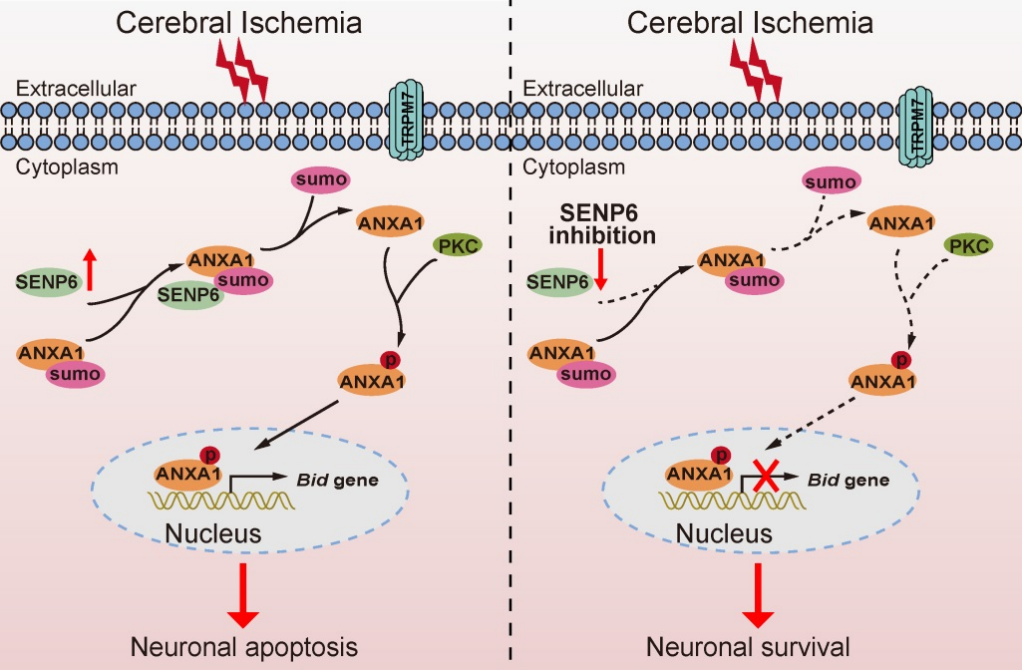
** Schematic diagram depicting strategies to protect against neuronal cell apoptosis induced by ischemic stroke via SENP6 inhibition.** The increase in SENP6 induced by cerebral ischemia causes ANXA1 deSUMOylation, followed by enhanced ANXA1 phosphorylation, which promotes ANXA1 nuclear translocation, ultimately activating Bid expression and the caspase-3 proapoptotic pathway. After SENP6 inhibition, the SENP6-ANXA1-p53-Bid-caspase-3 axis is inhibited, which eventually improves the survival of neurons subjected to cerebral ischemia-reperfusion injury.

In summary, our findings identified a previously unrecognized molecular mechanism by which SENP6 participates in neuronal apoptotic death after cerebral ischemia. More importantly, we have provided compelling evidence that SENP6 functions as an isopeptidase to deconjugate SUMO from ANXA1, thereby facilitating ANXA1 translocation to the nucleus, activating the caspase-3 apoptosis pathway and inducing neuronal apoptotic death after cerebral ischemia-reperfusion injury (Figure 9). To our best knowledge, this is the first study comprehensively describing the key roles of SENP6 mediated deSUMOylation of ANXA1 in regulating its nuclear translocation-associated neuronal apoptosis, and uncovering a novel pathophysiological mechanism of cerebral ischemia-reperfusion injury. Because lentivirus-mediated gene overexpression takes a certain amount of time *in vivo*, we pre-treated mice with lentivirus stereotactical injections two weeks before MCAO operation in this study, thus more detailed work will be needed to verify the therapeutic effect with a post treatment. On the other hand, systemically administration with an inhibitor which could selectively downregulate SENP6 enzyme activity or block its interaction with ANXA1 may provide protective effects against cerebral ischemia-reperfusion injury. Being the target a neuronal protein, its actual druggability may be also limited by the presence of the blood-brain barrier. Certainly, these hypotheses remain to be determined in future studies.

## Supplementary Material

Supplementary figures.

## Abbreviations

ANXA1: Annexin-A1
Co-IP: Co-immunoprecipitation
LDH: lactate dehydrogenase
MCAO: middle cerebral artery occlusion
mNSS: modified neurological severity score
MWM: Morris water maze
N2a: Neuro-2a
OGD/R: oxygen-glucose deprivation and reperfusion
PTMs: posttranslational modifications
qRT-PCR: Quantitative real-time PCR
SENP6: sentrin/SUMO-specific protease 6
shRNAs: short hairpin RNAs
SUMO: small ubiquitin-related modifier
TUNEL: TdT-mediated dUTP-X nick end labeling
TTC: 2,3,5-triphenyltetrazolium chloride
tPA: tissue-plasminogen activator
