# Supplementary material for: Inhibition of SENP6 restrains cerebral ischemia-reperfusion injury by regulating Annexin-A1 nuclear translocation-associated neuronal apoptosis
Source: Theranostics. 2021 Jun 1;11(15):7450–70. doi: 10.7150/thno.60277 (PMC8210613; doi:10.7150/thno.60277)
Supplement: Supplementary file 1 — Supplementary figures. [file thnov11p7450s1.pdf]

Supplementary Materials

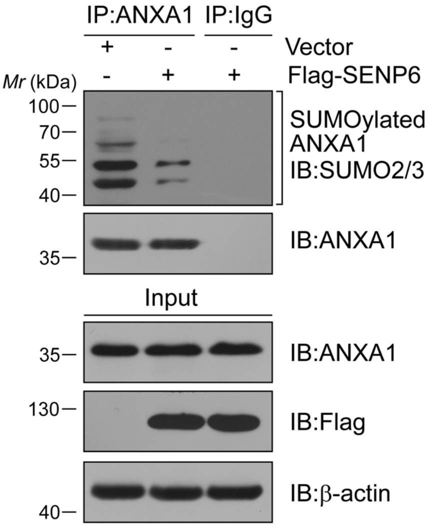

Figure S1. SENP6 decreased the SUMOylation level of ANXA1 in primary cultured microglia. Primary microglial cells were infected with adenoviruses expressing Vector or Flag-tagged SENP6. 48 h later, the cells were lysed and co-IP assay was used to determine the SUMOylation level of endogenous ANXA1. Data are representative of three independent experiments.

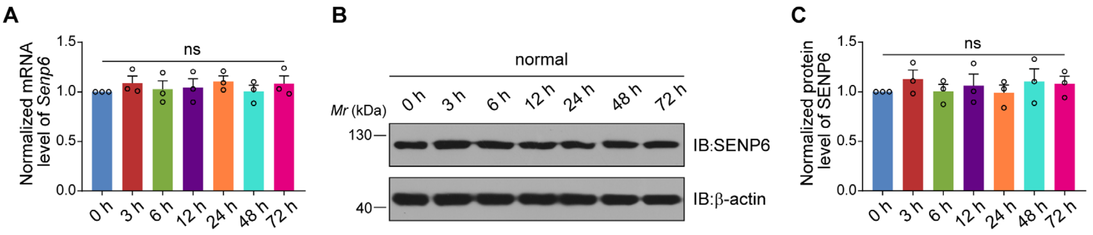

Figure S2. The temporal effect has little effect on the mRNA and protein expression of SENP6 under normal conditions.

(A) qRT-PCR was conducted to examine the *Senp6* mRNA level in primary cultured neurons at different time point under normal condition. The time point was corresponded with OGD/R treatment shown in Figure 1G. (B) Representative immunoblots showing the protein levels of SENP6 in primary cultured neurons at the indicated time points. (C) The quantitative analysis of SENP6 in (B). Data are reported as the mean ± S.E.M. from three independent experiments and analysed by one-way ANOVA followed by Dunnett's post hoc test. ns, no significance.

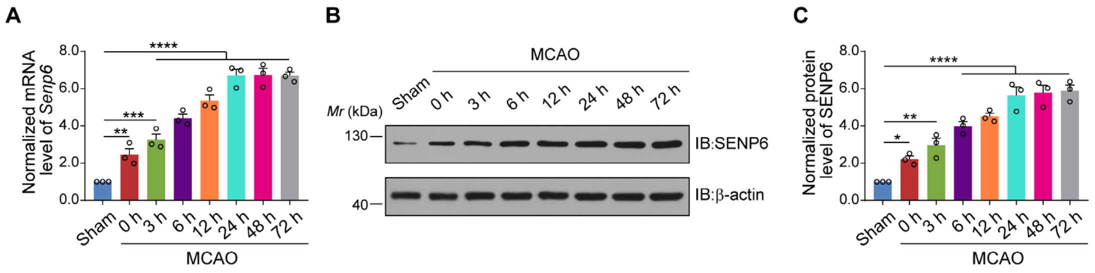

Figure S3. SENP6 was upregulated after cerebral ischemia-reperfusion injury in vivo.
(A) MCAO increased the mRNA and protein level of SENP6. qRT-PCR was conducted to examine the *Senp6* mRNA level. (B, C) MCAO increased the protein levels of SENP6. Mouse brain homogenates were extracted at the indicated time points following cerebral ischemia-reperfusion injury. Western blot assay (B) and quantification analysis (C) showing the protein expression of SENP6; n=3 per time point. Data are reported as the mean ± S.E.M. and analysed by one-way ANOVA followed by Dunnett's post hoc test. **P* < 0.05, ***P* < 0.01, ****P* < 0.001, *****P* < 0.0001.

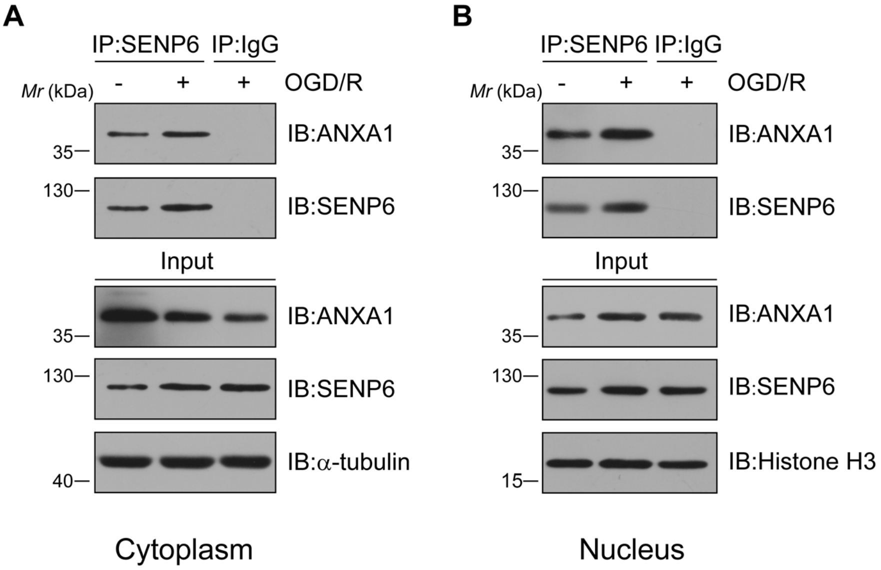

Figure S4. OGD/R enhanced the interaction between SENP6 and ANXA1 in the cytoplasm and nucleus fractionations.

(A, B) Representative co-IP results showing the endogenous interaction of SENP6 and ANXA1 in cytoplasmic and nuclear extracts of primary cultured neurons under normal and OGD/R conditions. The results are representative of three independent experiments.

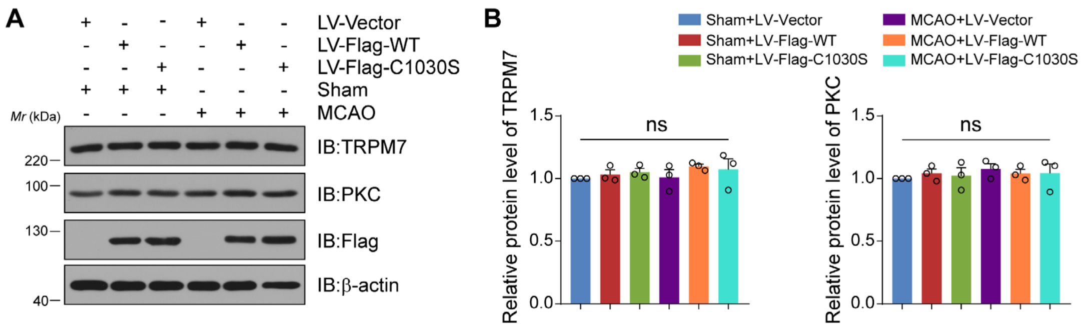

Figure S5. lentivirus-mediated overexpression of SENP6-WT or SENP6-C1030S had little impact on the protein level of TRPM7 and PKC in mice subjected to Sham or MCAO operation.

(A) Representative immunoblots showing the protein levels of TRPM7 and PKC in mouse brain homogenates. (B) The quantitative analysis of TRPM7 and PKC in (A), respectively. Data are reported as the mean ± S.E.M. from three independent experiments and analysed by one-way ANOVA followed by Dunnett's post hoc test. ns, no significance.

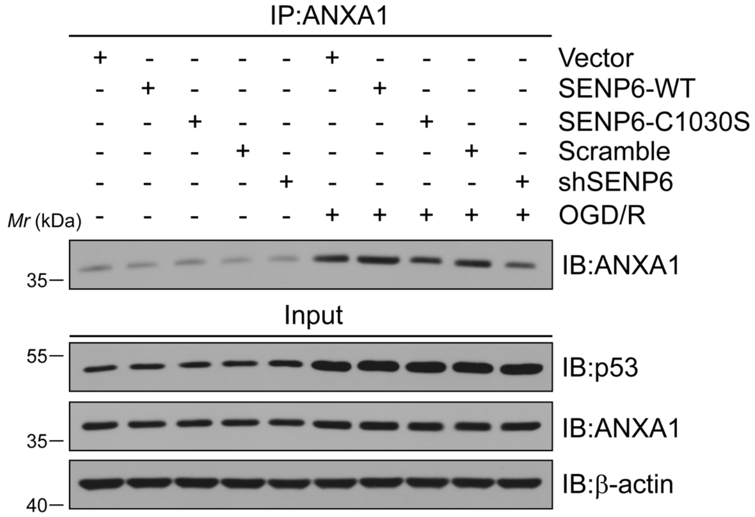

Figure S6. The overexpression or knockdown of SENP6 affected the binding of ANXA1 with p53 after OGD/R treatment, but with little effect on this binding at normal conditions. Representative co-IP results showing the endogenous interaction of ANXA1 and p53. Primary cultured neurons were infected with adenoviruses expressing Vector, Flag-tagged SENP6-WT, Flag-tagged SENP6-C1030S, shRNA against SENP6 or scrambled control, and the cell lysates were subjected to immunoprecipitation with an anti-p53 antibody, followed by immunoblotting analysis with an anti-ANXA1 antibody. Data are representative of three independent experiments.

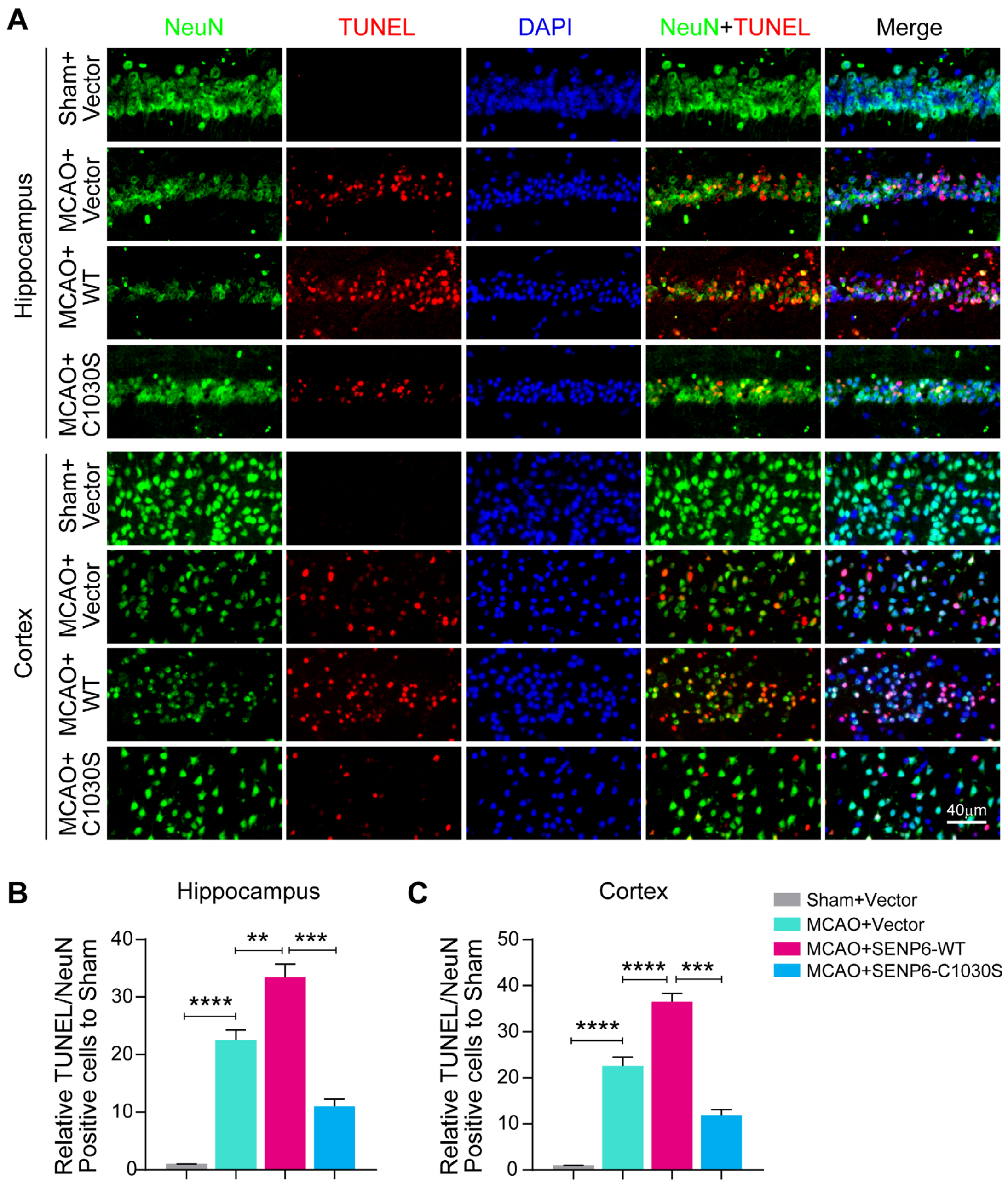

Figure S7. Inhibition of SENP6 restrained neuronal cell apoptosis induced by cerebral ischemic-reperfusion injury in vivo.

(A) Representative hippocampal and cortical sections from Sham+Vector, MCAO+Vector, MCAO+SENP6-WT, and MCAO+SENP6-C1030S-treated animals were subjected to double staining for NeuN and TUNEL. (B, C) Quantification was conducted by counting the number of NeuN-positive neurons and TUNEL-positive neurons. For all graphs, data are reported as the mean ± S.E.M. and analysed by one-way ANOVA followed by Dunnett's post hoc test. n = 5 mice per group. **$P < 0.01$, ***$P < 0.001$, ****$P < 0.0001$.
